# Bacteriome genetic structures of urban deposits are indicative of their origin and impacted by chemical pollutants

**DOI:** 10.1038/s41598-017-13594-8

**Published:** 2017-10-16

**Authors:** Romain Marti, Céline Bécouze-Lareure, Sébastien Ribun, Laurence Marjolet, Claire Bernardin Souibgui, Jean-Baptiste Aubin, Gislain Lipeme Kouyi, Laure Wiest, Didier Blaha, Benoit Cournoyer

**Affiliations:** 1 Research Group on “Bacterial Opportunistic Pathogens and Environment”, UMR Ecologie Microbienne, CNRS 5557, INRA 1418, UCBL, Université de Lyon, VetAgro Sup, Marcy L’Etoile, France; 2 DEEP, INSA Lyon, Villeurbanne, France; 3 Institut des Sciences Analytiques, UMR CNRS 5280 Villeurbanne, France

## Abstract

Urban activities generate surface deposits over impervious surfaces that can represent ecological and health hazards. Bacteriome genetic structures of deposits washed off during rainfall events, over an urban industrial watershed, were inferred from 16 S rRNA gene (*rrs*) sequences generated by high throughput sequencing. Deposits were sampled over a 4 year-period from a detention basin (DB). Major shifts, matching key management practices, in the structure of these urban bacteriomes, were recorded. Correlation analyses of *rrs* similarities between samples and their respective concentrations in chemical pollutants, markers of human fecal contaminations (HF183) and antimicrobial resistances (integrons), were performed. Harsher environmental constraints building up in the older deposits led to an increase number of *rrs* reads from extremophiles such as *Acidibacter* and *Haliangium*. Deposits accumulating in the decantation pit of the DB showed an increase in *rrs* reads from warm blooded intestinal tract bacteria such as *Bacteroides* and *Prevotella*. This enrichment matched higher concentrations of *Bacteroides* HF183 genotypes normally restricted to humans. Bacteriomes of urban deposits appeared good indicators of human-driven environmental changes. Their composition was found representative of their origin. Soil particles and rain appeared to be major contributors of the inferred bacterial taxa recovered from recent deposits.

## Introduction

Cities are divided into patches favoring commercial, industrial, residential, and recreational activities. A major feature of these patches is the presence of impervious surfaces including buildings and roads that will modify the urban drainage of runoffs and generate various environmental risks including flooding and transfer of pollutants into natural ecosystems^1^. In fact, impervious surfaces prevent infiltration of storm waters into local soil layers, and reduce a recharge of aquifers. This increases water flow and generates greater erosion and shearing forces that will contribute at mobilizing pollutants such as chemicals (metals, organic compounds) or biological (bacteria, viruses) ones^2,3^ often adsorbed on particles^4^. Re-suspended urban deposits transported by runoffs can impact the connected downstream systems by modifying their biological diversity and ecological equilibria^5,6^. In order to reduce their impact, several solutions have been proposed. Among them, the implementation of detention basins appeared one of the most attractive. These infrastructures accumulate runoffs collected by storm water drainage systems, and can trap the re-suspended deposits. They can thus reduce surface erosion and transfer of pollutants. Detention basins can trap large quantities of urban deposits, and several studies have investigated their properties in order to improve their efficacy^7^. These basins are connected to infiltration systems that will favor transfers of their water content into the connected aquifers. The vadose zone of these systems will trap hydrophobic pollutants.

Chemical and microbial components of urban deposits can be used to infer their origin but also as indicators of pollution levels over urban catchments. Several studies investigated their chemical composition, and showed significant concentrations of PAH (Polycyclic Aromatic Hydrocarbons), heavy metals and pesticides^1^. These confirmed the significant occurrence of petrol combustion by-products but also of chemicals from industrial and domestic activities such as alkylphenols (used as precursors of detergents or fuel additives), and PBDE (PolyBrominated Diphenyl Ethers, volatile bromine compounds) used to fireproof plastics and textiles^8^. These pollutants could drive modifications of natural microbiota because of their toxicity or be used as Carbon-sources for microbial growth. They could also be correlated with the release of exogenous and hazardous microbial contaminants. In fact, domestic wastes among urban settings are often contaminated by human and animal associated bacteria including pathogens such as *Salmonella typhimurium*, *Pseudomonas aeruginosa*, *Listeria monocytogenes*, and *Staphylococcus aureus* ^9–11^. Rain events could wash-off these bacteria from garbage sites and favor their dissemination. However, very few studies demonstrated the ability of such exogenous bacteria at surviving in urban deposits. Nonetheless, a significant contamination by fecal bacteria was reported^1^. Indeed, *Escherichia coli* was detected at concentrations as high as 4.6 log CFU g^−1^ of dry urban deposits. While there are very few data on the effect of urban pollutants on microbial diversity, some studies on agricultural soils revealed some significant microbial diversity changes. In fact, an increase in pesticide concentrations was recently correlated to an increase of IncP plasmids among a purification system containing soil microbiota^12^. These plasmids can harbor degradation genes but also antibiotic and virulence ones^12^. Similarly, an application of copper sulfate in a soil, during 5 years, led to an increase of Firmicutes^13^. Such a change in diversity was related to higher natural Cu resistance levels. The BASAMID® fumigand also led to a loss of bacterial ammonium oxidizers activity, and a decrease of bacterial operational taxonomic unit abundance levels^14^.

The above observations led us to hypothesize that urban deposits from a catchment should (1) harbor native soil microbial taxa but also exogenous ones representative of the human activities occurring over an urban area; (2) have selected microbial taxa adapted for a use or detoxification of urban chemical pollutants; (3) have selected microbiota adapted for a survival and development in deposits that have settled among detention basins, and been exposed to strong environmental constraints including hydrological forces. To address these issues, a Next Generation Sequencing (NGS) 16 S rRNA gene (*rrs*) analysis of accumulated urban deposits among a detention basin was performed. This approach allows reliable bacterial classifications down to the genus level^15^. The OTHU (Lyon Field observatory of urban hydrology) detention basin (DB) was selected for this study. This basin, hereafter named Django-Reinhardt (DjR), receives drainage water from a 185-hectare urban industrial basin that is 75% impervious. The basin covers one hectare of surface area and has a maximum volume of 32 000 m^3^. It is connected to an infiltration basin. This is the world’s most common type of basin^16^. OTHU performs a long term monitoring of the DjR site including water volumes being collected during rain events, flow values, and also physico-chemical parameters of the inflows and outflows such as pH, electrical conductivity, and turbidity^2^. Grab samples have also been analyzed to monitor chemical pollutants. From 2010, microbial analyses of the DjR sediments have been undertaken. This work presents the first NGS *rrs* datasets from urban deposits. Investigation of statistical relations between bacteriome *rrs* genetic structures, chemical pollutants, bacterial indicators such as integrons, and physico-chemical parameters of these deposits required the use of a nested sampling design. This scheme increased the likelihood of identifying key explanatory variables. Class 1, 2 and 3 integrons were estimated because of their previously described biased distribution among bacteria. These integron classes are plasmid-borne, and often harboring antibiotic resistance genes^17,18^. Presence of class 1 integron was considered evidence of human-driven environmental changes such as those related to releases of fecal matter and pharmaceuticals^19^. DNA extracts for NGS were recovered from several sites over the DjR detention basin in order to better resolve the variances associated with such datasets. Samplings were performed from 2010 to 2014. The NGS *rrs* genetic structures were compared between sampling sites, sampling years, and correlated to site maintenance practices, and hydrological and physico-chemical datasets.

## Results and Discussion

### Sampling campaigns

**Figure 1:**
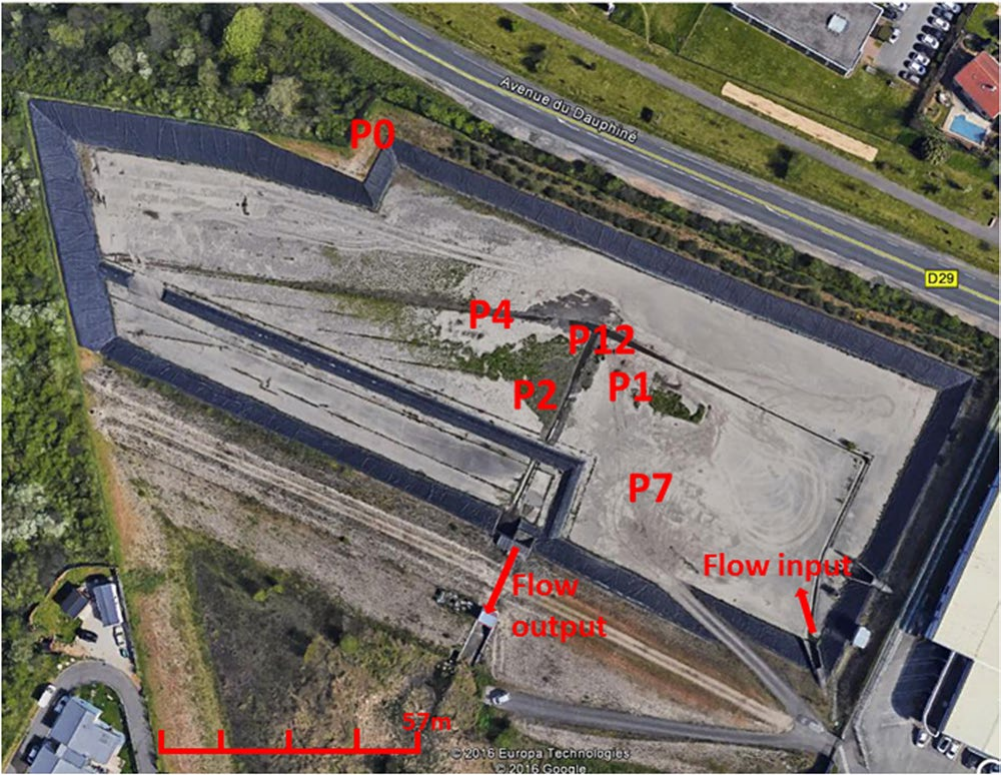
Numbered position of each sampling site (P) of the Django Reinhardt detention basin (DjR DB) of the “mi-plaine” industrial zone of Chassieu (France). Red arrows indicate the runoff entry point, and outflow of the detention basin. The outflow is directed toward an infiltration basin connected to the Lyon east aquifer. P0 was the control site located outside but at the edge of the detention basin. P12 is a decantation pit. Recent deposits can be observed in the DB.

Field sampling campaigns and sites are presented in Fig. 1 and Table S1. Measured field parameters suggested the 2012 (71-month accumulation period) sampling campaign of the DB to be unique in terms of rain volume and intensity, one week prior to sampling, with values much higher (33 mm and 266 mm h^−1^ respectively) than those of the other periods (Table S1 to Table S10 in the supplementary materials). The 2010 (54-month accumulation period) and 2013/2014 (6 to 15 month accumulation periods) campaigns showed similar rainfall regimes prior sampling (Table S1). Main differences between these campaigns were the sampling season, and the time period from the last date of total removal of the sediments from the detention basin. Sediments had been totally removed in the first months of 2006 and in March 2013. Principal component analysis of the general features describing the deposits grouped most samples recovered from the surface of the basin, and differentiated P12 (decantation pit) and P0 samples (outside but at the edge of the DB) (Fig. S1). The P12 samples from February, April and July 2014 were clearly apart from the other samples based on their granulometry, as observed in Table S4. These higher particle sizes observed at P12 indicated a strong incidence of the decantation pit on this parameter. Total Carbon (C-total), total Nitrogen (N-total) and total Phosphorus (P-total), and water content of the deposits at sampling time were significantly different between P0 (control) and DB samples (Tables S3 and S7).

### Chemical profiling of urban deposits

PAH and MTE (metallic trace elements) contents of the deposits were measured. PCA were performed on these datasets (Fig. S1a). The P0 site, on the upper edge of the detention basin, was found harboring significant chemical pollutants coming from the road and sidewalks surrounding the detention basin. PCA of the concentrations of 16 types of PAH identified patterns among the sampling sites (Table S8). Tracking of these PAH is recommended by the European Food Safety Authority (EFSA) and Environmental Protection Agency (EPA, USA), due to their carcinogenicity or genotoxicity. PAH patterns from site P12 samples from the decantation pit were grouped (with the exception of the April 2014 sample) in a time independent manner. These P12 samples showed PAH patterns quite different from those of the other sites, with a strong positive relation with naphthalene (H1) concentrations and negative ones with other PAH (Fig. S1a). Naphthalene is toxic for several bacterial species but some can use it as a C-source^20^. It can thus trigger changes among a bacterial community. PAH patterns of the 2014 February and April samples from the P1, P2, P4 and P7 sites appeared closely related. Those of July 2014 were well differentiated from those collected at the other dates from the same sites. They had profiles closer to those of site P12. October 2013 samples showed a distribution around the central point of the PCA, indicating a weak relation with PAH concentrations. PAH patterns and concentrations among the DjR basin thus appeared to have evolved rapidly with the outdoor conditions. This was previously described^1^. In a previous study, the P12 deposits were found more toxic than samples from the other sites. Still, the PAH profile related to this particular sampling campaign was atypical. High PAH concentrations had been recorded in 2012 while low values were observed for the following years except for naphthalene (Fig. S1). Still, this suggested that such contaminants are likely to impact the bacteriome of these urban deposits. The [phenanthrene/anthracene (Ph/A) and fluoranthene/pyrene (F/Py)] ratios were computed in order to infer their origin^21^. Ratios <10 and >1 for Ph/A and F/Py respectively are typical of pyrogenic combustion, and >10 and <1 of fuel combustion^22^. The following samples showed pyrogenic combustion signatures for both indicators: (1) the P1, P2, P4 and P7 2012 samples, (2) the P1, P4 and P7 samples of February 2014, and (3) the April 2014 P1 and P7 samples. Other samples had petrogenic combustion signatures. The Ph/A ratios were high for (1) P0 samples, (2) all P12 samples except the ones of April and July 2014, and (3) all October 2013 samples except the one recovered at P2. Low F/Py ratios were observed for (1) all July 2014 samples (P0 to P12), (2) P2, P4, and P12 samples of April 2014, (3) P2 in February 2014, (4) P1, P2, and P4 in October 2013. These datasets indicate that both pyrogenic and fuel combustions impacted the area.

The distribution patterns of metallic trace elements (MTE) (Table S9) appeared more straightforward than those of the tracked PAHs. Similar trends were observed between the 2010 and 2012 MTE datasets. Higher concentrations for all MTE except Zn were observed for the 2010/2012 period than the ones of 2013/2014 (Table S9). A relation between accumulation time periods and MTE concentrations was thus observed. Concentrations measured in July 2014 were the lowest except for Zn. Only discrepancies in these trends were observed when comparing P12 with the other sampling sites. Cu and Pb showed significantly lower concentrations at P12 than elsewhere in the basin. It is to be noted that Cr and Ni concentrations were not available for the 2010 P1, P2, P4, and P7 samples. All MTE measurements were not available for the P12 2010 samples.

### Integron and Bacteroidales contents of urban deposits

Numbers of class 1, 2 and 3 integrons, and of 16 S rRNA gene from Bacteroidales HF183 and total Bacteroidales cells were estimated between sampling sites and dates. These datasets are shown in Table S10 and were analyzed by PCA (Fig. S1b). October 2013 P12 and P4 sampling sites showed the highest ratios of *int2* and *int3* contents. All February 2014 samples but the P2 one also showed significant *int2* and *int3* contents. P12 April 2014 and P12 2012 samples had relatively higher *int1* contents. The numbers of *int* copies were found related to high numbers of Human Bacteroidales HF183 or total Bacteroidales markers (Fig. S1). These variations indicate significant changes in the bacteriome genetic structures over time and between sampling sites. Detection of Human Bacteroidales HF183 indicated significant contaminations by human feces^23^, and these appeared to have occurred whatever the season. A study of fecal contaminations over the watershed would be required in order to identify the sources. Detection of *int* genes was less surprising than the one of the human fecal marker. However, *int3* showed a prevalence of 100% among the urban deposits. So far, integrons of class 1 were considered more prevalent than those of classes 2 and 3^17^. It thus appears that an industrial watershed could enrich *int3* bacteria. Integrons can encode genes involved in antibiotic and MTE resistances. The class 3 integron has been associated with soil and freshwater bacteria such as *E. coli, P. aeruginosa*, *P. putida*, *Klebsiella pneumoniae* or *Salmonella* spp. but evidence of a strong linkage with antibiotic resistances is lacking^18^. It has, sometimes, been found harboring gene cassettes encoding beta-lactamases such as *bla*_GES-1_^19^. An industrial watershed might select integrons involved in the degradation properties of urban contaminants rather than contributing at the resistances towards antibiotics. Pharmaceuticals were not monitored in this study. Nevertheless, they are likely to be found because of the fecal contaminations observed over the watershed. Human fecal matter is an indicator of their presence^24^. However, their concentrations are expected to be low.

### 16S rRNA gene inferred bacterial genetic structures of urban deposits

V5-V6 16S rRNA gene PCR products were produced from DNA extracts of all sites of the detention basin including P0. Sequencing of the 2010/2012 PCR products led to 154,625 V5-V6 sequences with 8,727 to 17,942 sequences obtained per sample. Sequencing of the 2013/2014 PCR products led to 1 210,587 sequences with 10,119 to 91,749 sequences per sample. These datasets were merged, and the number of V5-V6 16 S rRNA gene sequences per sample was normalized to 8,727 (for a total of 305,445 sequences). From the normalized database, 27,985 OTUs (operating taxonomic unit defined for each group of identical reads) were recovered. The most numerous OTU yielded 5,006 sequences (1.6% of the total) and was related to KCM-B-112 family of the Gamma-Proteobacteria. This OTU was first recovered from a soil near a non-ferrous metal smelting factory named KCM localized in Bulgaria (see accession number AJ581597). Overall, the 100 most abundant OTU showed number of reads above 400. Rarefaction curves of OTU numbers showed asymptotes for all samples from the detention basin (Fig. S2). This indicates the recovery of a significant part of the diversity which can be revealed by the V5-V6 16 S rRNA gene sequences. P0 samples, from outside the basin, showed a less pronounced plateau. The higher diversity observed at the P0 sampling point suggests a more favorable environment (lower constraints) for bacterial survival. This is in agreement with our hypothesis that urban deposits (in a detention basin) are more noxious and thus more selective.

**Figure 2:**
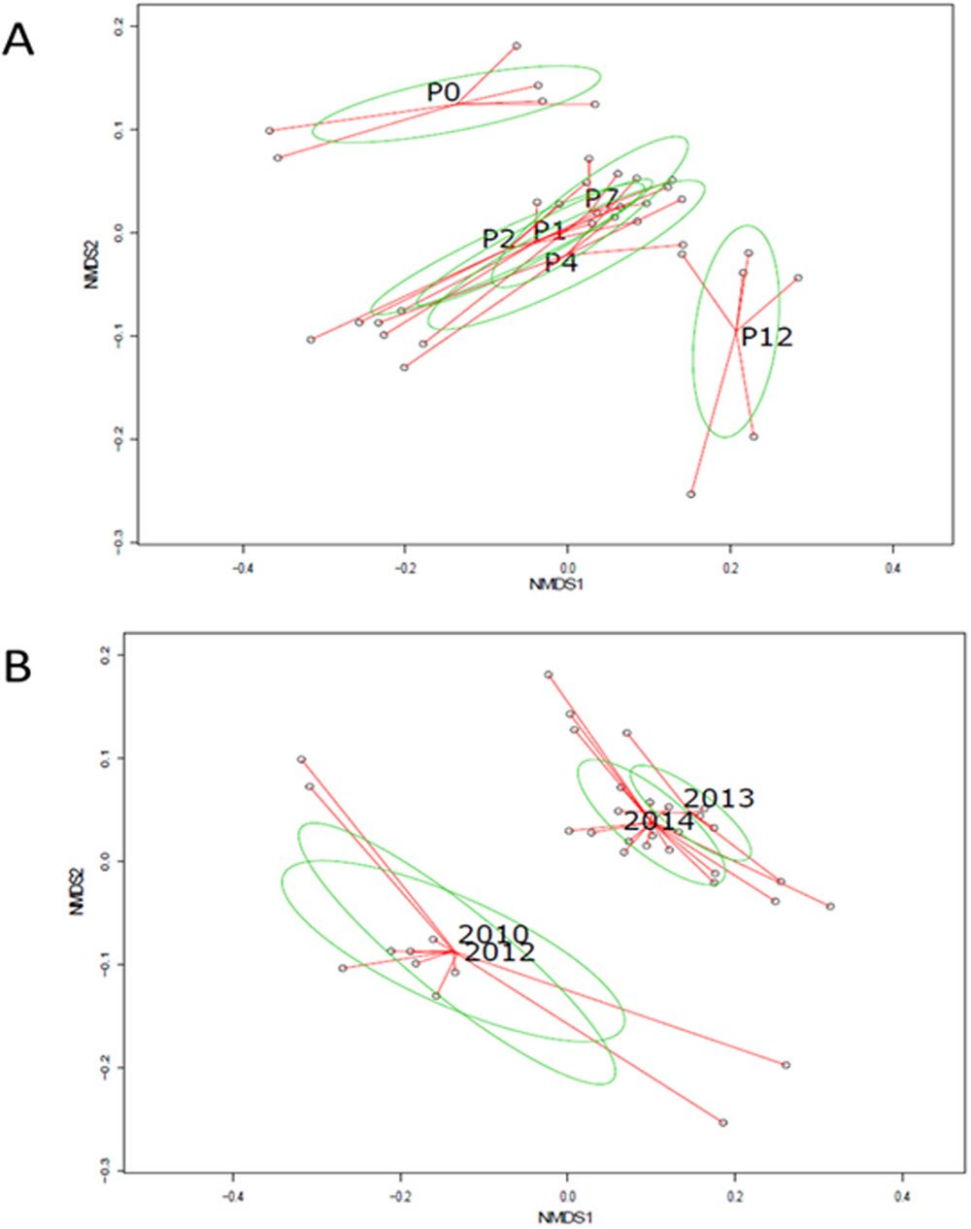
NMDS plotting of Bray-Curtis dissimilarities computed from the number of 16 S rRNA gene reads per OTU (identical sequences) recovered from the sampled urban sediments. Sediments were sampled at sites P0, P1, P2, P4, P7 and P12 between 2010 and 2014. See Fig. 1 and Table S1 for more details on the sampling campaigns. Stress test value was 0.08. The green ellipses are representative of the variance observed (standard error) in the ordinations per group of samples (**A**) per site and (**B**) per year. AMOVA tests confirmed the significance (p < 0.01) of the main groupings when the ellipses were not superimposed. On (**A**), datasets from a same site are linked by red lines drawn from the centroid of the confidence ellipse, and on (**B**) datasets from a same sampling year are linked by a red line drawn from the centroid of the confidence ellipse.

The OTU contingency table was used to compute a Bray Curtis dissimilarity matrix, and build a dendrogram illustrating the relationships between samples (data not shown). This dendrogram was compared to NMDS plotting of the Bray Curtis dissimilarities. The Bray Curtis dendrogram led to the observation of four large groups of OTU profiles: (1) one for the P0 samples, (2) one for those of the P12 site, (3) one for the 2010 and 2012 samples without P12, and (4) one for the 2013 and 2014 samples without P12. This is in line with the NMDS plots shown in Fig. 2. Unweighted and weighted Unifrac tests were performed to test the reliability of the Bray Curtis dendrogram according to (1) the sampling sites, and (2) the sampling years. Unweighted (qualitative metric) UniFrac tests detected no significant relationship (p > 0.05) between the groupings and sites but showed the 2014 samples to have a significant tendency at being clustered (p < 0.01) apart from the other samples. Weighted (quantitative metric considering the number of reads per OTU) UniFrac showed significant relationships between the groupings (p < 0.01). Segregation of the 2013 and 2014 samples from the others was also supported by significant P-values (p < 0.01). NMDS plotting also showed P0 and P12 samples to have distinctive OTU profiles, apart from those of P1, P2, P4 and P7 (Fig. 2A). A Permutation test with p < 0.05 and AMOVA with p < 0.001 confirmed the significance of these differences between sites. This is also in line with the segregations inferred from the chemical distribution profiles (Fig. S1) where P12 had distinctive contents, respectively, in terms of C-total and other potential nutrients, and PAH. NMDS ordinations of OTU patterns also matched a differentiation according to sampling years (Fig. 2B). The OTU profiles of 2010 and 2012 were apart from those of 2013 and 2014. A permutation test with p < 0.001 and AMOVA with p < 0.001 confirmed the significance of these differences. This segregation matches the distinction between an accumulation over at least 54 months, and sampling years where dredging had occurred recently (less than 15 months). A maturation of the sediment over time associated with a selection of particular bacterial species likely explains these differences. In order to test the effect of hydro-dynamism on the bacteriome of urban deposits, an additional NMDS analysis was performed by limiting the dataset to the P1 to P7 sites. However, the stress value associated with this analysis was nearly zero indicating a weak variability between these sampling sites (data not shown). However, limiting this analysis to the 2013/2014 datasets revealed a significant differentiation of the P2 and P7 sites with some overlaps with the P1 and P4 ones. These observations suggested an impact of the DB hydro-dynamism on the V5-V6 *rrs* diversity. Yan *et al*.^7^ indicated the high flow turbulent kinetic energy near the bottom of the DB to be the main factor impacting the accumulation of deposits in the DjR detention basin. This was confirmed in this study with clear biases in the distribution patterns of the deposits indicating such forces to be greater at P7. In 2010, the thickness of the deposit layer was greater for P4 > P2 > P1 > P7^1^.

### Taxonomic allocation of 16 S rRNA gene OTU and definition of urban core bacteriomes

**Figure 3:**
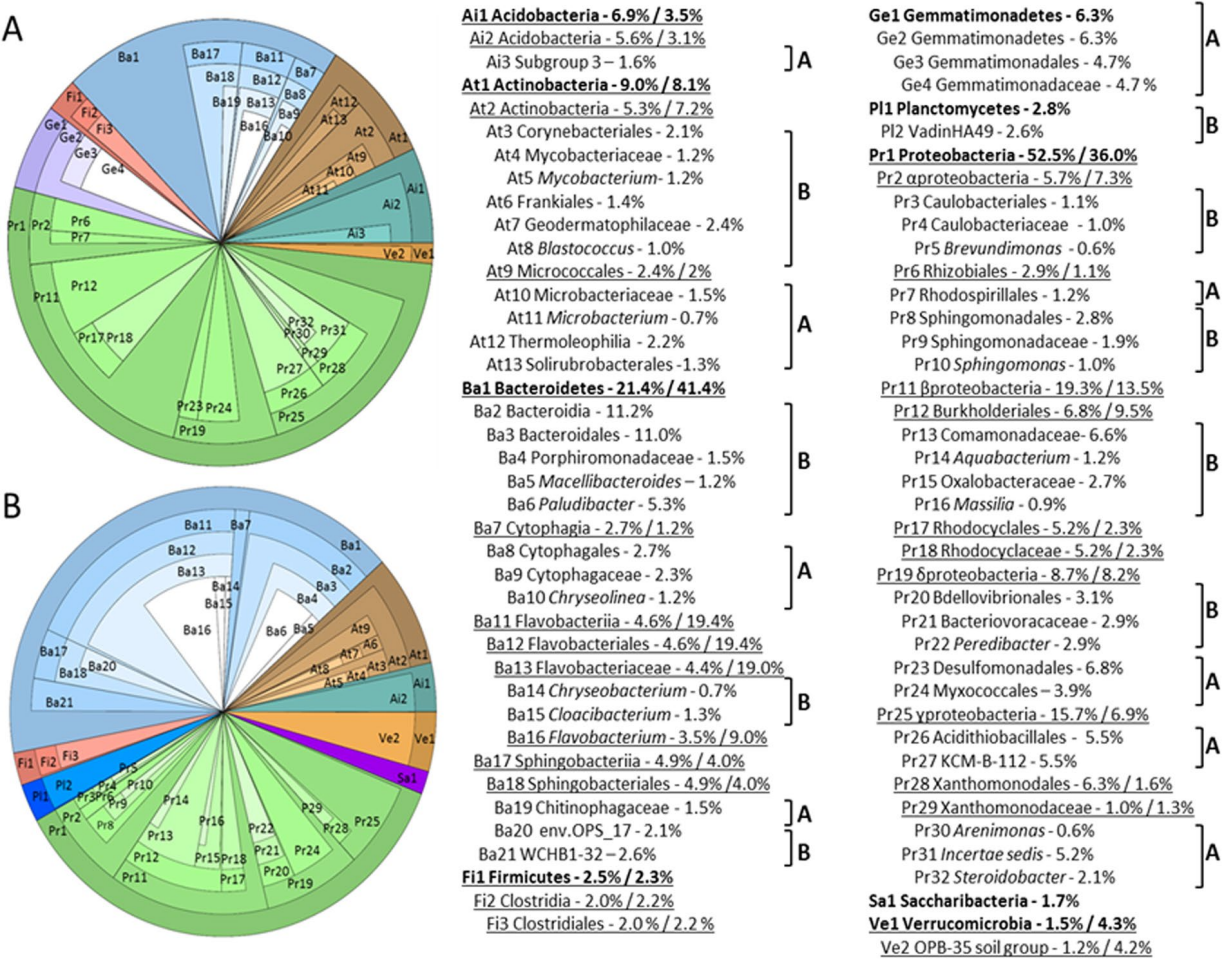
Core bacteriomes of urban deposits recovered from a detention basin and inferred from the taxonomic allocation of 16 S rRNA gene reads. (**A**) Main bacterial groups of deposits which accumulated from beginning 2006 and were sampled in 2010 (54-month accumulation period) and 2012 (71-month accumulations); (**B**) main bacterial groups of deposits which accumulated from march 2013, and were sampled from October 2013 (6-month accumulations) until July 2014 (15-month accumulations). Only taxa representing more than 1% at the family level and/or 0.5% at the genus level are represented. Phyla are in bold. Most common phylogenetic groups are underlined. Brackets highlight the distribution of specific taxa among each bacteriome (according to their above letter code). Relative proportion of the groups among each 16 S rRNA gene dataset is indicated. Classification goes from the external part of the pie towards its center.

Phylogenetic allocations of the above OTU recovered from urban deposits were performed by comparison with the Silva bacterial 16 S rRNA gene database (v123). Two core bacteriomes (Fig. 3) were defined according to the biases observed on the NMDS plots (Fig. 2), with (a) considering the 54–71 month deposits accumulation periods (2010/2012), and (b) the “recent” 2013/2014 ones (6–15 months periods). Cutoffs of 1% and 0.5% over the total number of reads were used to select, respectively, families and genera to be shown on the pie charts (Fig. 3). These bacteriomes shared 6 phyla: Acidobacteria, Actinobacteria, Bacteroidetes, Firmicutes, Proteobacteria and Verrucomicrobia, but discrepancies were observed in terms of proportion of reads per phylum. Mature sediments (2010/2012) showed lower numbers of Bacteroidetes than fresh ones (2013/2014) (21.4% against 41.4%) but had more Proteobacteria (52.5% in 2010/2012 against 36.0% in 2013/2014). Gemmatimonadetes were at numbers sufficient to appear on the 2010/2012 core bacteriome, but not the Planctomycetes and Saccharibacteria which appeared only on the one of the 2013/2014 dataset (Fig. 3). Combinations of phyla among these core bacteriomes were compared with those from river and estuarine sediments that were exposed to similar chemical pollutants. PAH-impacted estuarine sediments were also dominated by Actinobacteria (from 15 to 41% according to depth), Proteobacteria (15 to 42% - especially the Alpha-Proteobacteria) and Firmicutes (2 to 30%)^25^. Urban river sediments impacted by high MTE and organic matter (e. g. from a wastewater treatment plant or combined sewer overflows) were dominated by Proteobacteria (34 to 46%), Bacteroidetes (7 to 15%), and Firmicutes (16 to 22%) but also significant numbers of Planctomycetes (7 to 17%)^26^. Proteobacteria were also found to be the dominant forms in peri-urban river sediments impacted by human activities followed by either the Bacteroidetes or Firmicutes^15,27,28^. A similar situation was observed for a river contaminated by wastewaters from unconventional oil and gas treatment facilities^28^. Nevertheless, similar phyla, and respective relative proportions, were observed among less impacted sites such as the source of a river from a rural environment^15^. Thus, independently of the investigated ecosystem, the main bacterial phyla of urban deposits appeared to be similar to those from natural river systems. However, old deposits appeared to have undergone competitive exclusion according to Gauze’s law^29^ which led to a significant decrease in the number of reads for phyla such as Saccharibacteria and Planctomycetes. However, these trends did not change the diversity indices computed at the phyla scale (data not shown).

**Figure 4:**
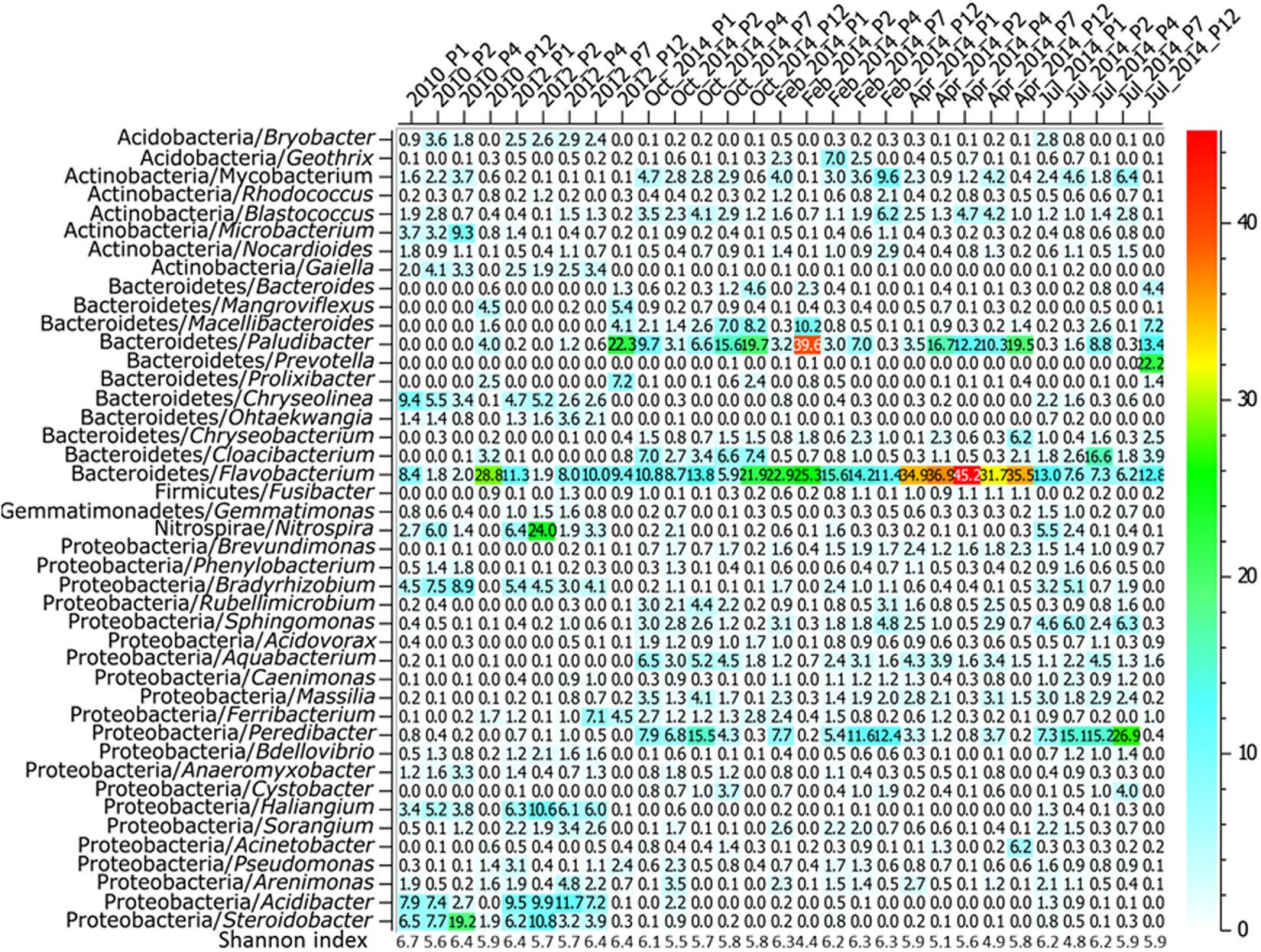
Heat map showing the relative abundance of the most frequent bacterial genera among urban deposits inferred from 16 S rRNA gene reads. Data are given considering sampling periods and sites. A 0.45% total relative proportion in the dataset was used as a cutoff (over 43 genera). Shannon diversity indices are given at the bottom. See Fig. 1 and Table S1 for more details on the sampling campaigns. Scale on the right gives the color code for the relative occurrences.

The 2010/2012 core bacteriome showed Proteobacteria to be dominated by *rrs* reads from the Beta-proteobacteria (19.3%), followed by those of the Gamma-Proteobacteria, Delta-Proteobacteria and Alpha-Proteobacteria (respectively 15.7, 8.7 and 5.7% of the reads). Bacteroidetes *rrs* reads were divided into three highly significant classes, with proportions going from 2.7, 4.6 and 4.9%, respectively, for those of the Cytophaga, Flavobacteria and Sphingobacteria. *Microbacterium* (0.7%, significantly higher in the 2010 dataset, Fig. 3), *Chryseolinea* (1.2%), *Flavobacterium* (4.4%), *Arenimonas* (0.6%) and *Steroidobacter* (2.1%) were significant components of this bacteriome. The 2013/2014 core bacteriome showed high content of reads from the Flavobacteria which represented 19.4% of the reads from the Bacteroidetes. This is almost 5 times the number of reads observed in the 2010/2012 dataset. *Flavobacterium* spp. are commonly found in sediments^15^. Among this bacteriome of recent deposits, reads from the Beta-proteobacteria (13.5%) were also the highest among the Proteobacteria. Twelve genera showed a higher number of *rrs* reads in these more recent deposits (2013/2014 dataset, see Fig. 4): *Mycobacterium* (1.2%, 2012 distribution was significantly different from the others,) and *Blastococcus* (1.0%) from the Actinobacteria, *Macellibacteroides* (1.2%; 2010 distribution was significantly different from the others), *Paludibacter* (5.3%, October 2013 set was significantly different), *Chryseobacterium* (0.7%; 2010/2012 patterns were significantly different from the others) and *Cloacibacterium* (1.3%; October 2013 set was significantly different) of the Bacteroidetes, *Brevundimonas* (0.6%), *Sphingomonas* (1.0%), *Aquabacterium* (1.2%), *Massilia* (0.9%) and *Peredibacter* (2.9%) of the Proteobacteria. Genera (n = 43) among the samples, occurring in a proportion over 0.45%, are shown in Fig. 4. From this contingency table, genetic diversity indices were computed, and did not reveal significant variations between OTUs from recent and mature deposits (Fig. 4).

### Spatial distribution biases of inferred bacterial genera

Shannon indices computed from the genus contingency table were compared between sampling sites (Fig. 4). Comparisons of indices between P2 and P1 samples, and P2 against P12 samples showed significant differences (p = 0.008 and 0.039, respectively). P1 and P2 are separated by a gutter while P12 is a decantation pit. These organizations thus impacted the inferred bacterial genetic diversity. P2 had the lowest values (Fig. 4). Occurrences of reads from *Prolixibacter* and *Anaeromyxobacter* showed spatial variations with a significant effect of the P12 site (Table S11). This site, the decantation pit, was previously shown to harbor deposits chemically distinct from those recovered from the surface (e. g. Fig. S1). Reads from the *Bacteroides* were also found to have a biased distribution pattern. *Bacteroides* were detected for every sampling campaign, with proportions up to 4.6% of the total number of reads. *Bacteroides* are indicative of fecal contamination^19^. Their presence is thus in agreement with the detection of significant numbers of the HF183 MST (Microbial Source Tracking) marker indicative of human fecal contaminations (Fig. S1). This genus is strictly anaerobic, and the distribution of its *rrs* reads was found to match (with a positive Spearman’s correlation rho factor of 0.84, p < 0.001) the one of reads allocated to *Paludibacter*, another genus grouping anaerobes. This correlation suggests a tropism for similar ecological niches between these genera. The presence of *Bacteroides* questions the sources of fecal pollution which led to their significant occurrence in the DB. Possibilities are: (i) unauthorized connections of sewer systems on the storm water network, and (ii) fecal contaminations from domestic and wild animals (and may be humans) living on the connected watershed. Fecal pollution is often associated with the occurrence of pathogens, and its detection thus suggests a risk for users of the groundwaters fed by the detention/infiltration basins investigated in this work. Selective bacterial platings of serially diluted DB deposits confirmed the presence of *Nocardia* spp., *Aeromonas* spp., and *P. aeruginosa* (data not shown). The development of DNA gene targets allowing taxonomic allocations at the species and sub-species levels will be required to further investigate the distribution patterns of these bacterial groups.

### Correlations between 16 S rRNA genetic structures and field variables including pollutants

**Figure 5:**
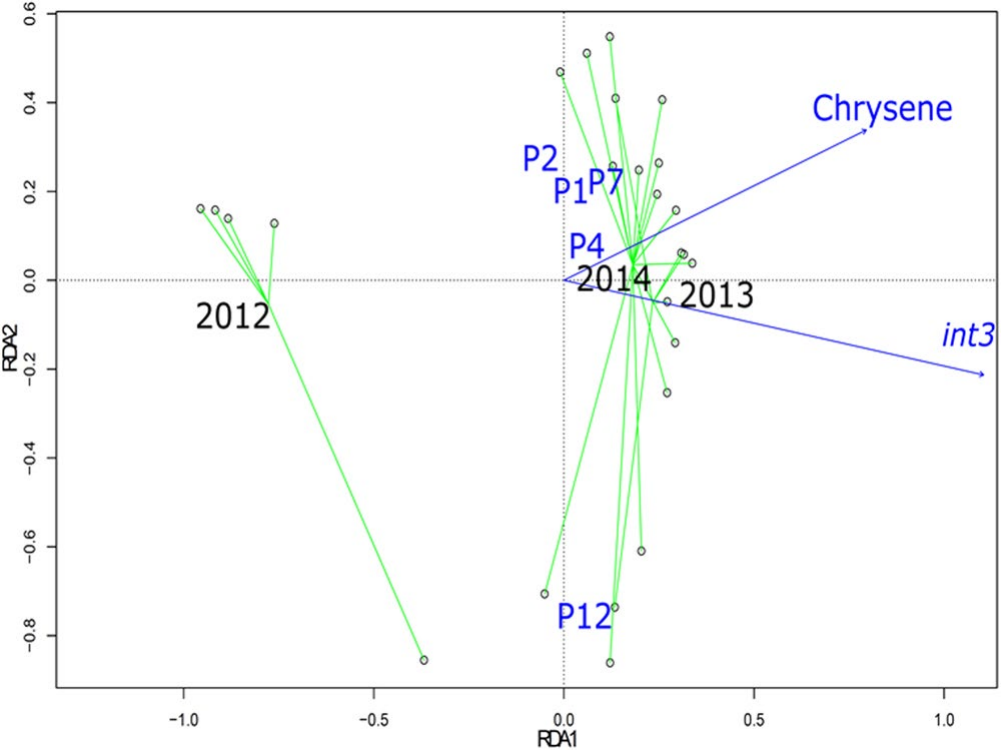
RDA (redundancy analysis) model inferred from urban deposits datasets. Ordinations of 16 S rRNA gene OTU datasets were constrained according to chrysene concentrations, *int3* ratios, sampling sites and years. Green lines are linking samples from a same year. See Fig. 1 and Table S1 for more details on the sampling campaigns.

Two approaches were used to investigate relations between the V5-V6 rRNA genetic structures and explanatory variables: (1) RDA (Redundancy Analysis), a direct gradient analysis technique which summarizes linear relationships between response and explanatory variables^30^, and (2) correlation tests between NMDS ordinations and measured values, associated with permutation tests to assess the significance of the observed relations. Results are summarized in Table 1. Figure 5 shows the RDA biplot relating *rrs* OTU patterns, sampling time and sites, and the explanatory variables.

**Table 1:** Significant relations highlighted by permutation tests from NMDS and RDA 16 S rRNA gene OTU ordinations plotted against explanatory variables*.

Type	NMDS	RDA	Variable strength in RDA
**MTE** ^§^			
Cd	+***	NA^#^	
Cr	+*	NA	
Ni	+*	NA	
Pb	+*	NA	
**Physical-chemical parameters**			
N	+*		
**PAH**			
Fluorene	+*		
Chrysene	+*	+**	2
Dibenzo(a,h)anthracene	+*		
**Molecular**			
*int3*	+*	+***	1
HF183	+**		
**Time and location**			
Year	+***	+**	3
Site	+*	+**	4

See Tables S1 and S2 for more details on measured parameters and sampling campaigns, + : a significant correlation between a variable and inferred ordinations. *p < 0.05, **p < 0.01 and ***p < 0.001. ^§^February and April 2014 MTE values were missing, ^#^NA: Not Applicable (missing values).

RDA could not be computed using the MTE [Cd, Cr, Ni, and Pb] datasets (Table S9) because of missing values. However, correlation tests could be performed using the NMDS ordinations of the full NGS *rrs* dataset. [Cd] showed the most significant correlation with the NMDS ordinations (r^2^ = 0.84, p < 0.01). [Cr, Ni and Pb] showed significant linear correlations but with a r^2^ ranging from 0.41 to 0.45 (p < 0.05). No significant correlation could be detected with the Cu and Zn values. Plots of [Cd, Cr, Ni, and Pb] over NMDS ordinations were drawn (Fig. S3), and showed similar distributions biases, with the highest concentrations being associated with the inferred 2010/2012 (mature deposits) OTU ordinations. MTE thus appeared to have accumulated over time. This accumulation matched the segregation of the dataset into two significantly different bacteriomes (Fig. 3). MTE were previously shown to drive bacterial community changes^31^. A significant incidence of [Cd] (34–134 mg kg^−1^) on a soil microbiota (fungal and proteobacterial groups) was observed by a DGGE DNA typing approach^32^. In our study, highest concentrations were of 8.5 mg kg^−1^ in 2010^1^.

For PAH, significant correlations were found between NMDS OTU ordinations (full dataset) and [fluorene] (r^2^ = 0.31, p < 0.05), [chrysene] (r^2^ = 0.25, p < 0.05) and [dibenzo (a,h) antracene] (r^2^ = 0.34, p < 0.05) (Fig. S4). RDA also identified significant correlations with [chrysene] (Table 1, Fig. 5). Highest [fluorene] concentrations were associated with the P12 samples (40 to 70 ng µg^−1^ of dry sediment instead of 10 to 30 ng µg^−1^ for P1, P2, P4 and P7) (Fig. S4A). Highest [chrysene] were associated with the 2013/2014 (recent deposits) OTU ordinations (50 to 70 ng µg^−1^ of dry sediment for 2013/2014 and 80 to 140 ng µg^−1^ of dry sediment for the 2012 samples) (Fig. S4B). Highest [Dibenzo (a,h) anthracene] (between 7 to 10 ng µg^−1^ of dry sediment) were found related to the P1, P2, P4 and P7 OTU ordinations. P12 samples had the lowest concentrations (6 to 7 ng µg^−1^ of dry sediment) (Fig. S4C).

PAH was previously associated with the presence of PAH degraders from the *Mycobacterium*, *Nocardioides*, *Sphingomonas*, *Paracoccus*, and *Pseudomonas* genera^33–35^. Significant numbers of *rrs* reads from the DB deposits were found allocated to these degraders (except P12 in July 2014 for *Nocardioides*). The highest relative number of reads for these degraders was 9.6% for *Mycobacterium* at the P12 site in February 2014, and appeared to match the [fluorene] distribution pattern which was highest in P12 samples. Regarding chrysene, its degradation was found to be carried by species such as *Alcaligenes faecalis, Mycobacterium parmense, Pseudomonas mexicana*, *Bacillus* sp., *Pseudomonas* sp. and *Paracoccus* sp.^36^. However, pairwise correlation tests did not identify any *rrs* OTU of the related genera fluctuating significantly with [chrysene]. Dibenzo (a,h) anthracene was previously shown related to changes in microbial community genetic structures^37^. In our case, a significant relation was observed with the NMDS ordinations but mainly related to the older deposits. The P12 sampling sites and all of those from the 2013/2014 datasets were associated with low concentrations of dibenzo (a,h) anthracene. One explanation could be that microbial consortia related to these samples were more efficient at using this molecule as a C-source.

Statistical tests were also performed with total - carbon, - phosphate and - nitrogen concentrations as the explanatory variables of the observed relations with the computed OTU patterns (Table S7), and bacterial qPCR DNA makers (Table S10). Nitrogen contents showed significant correlations with the NMDS ordinations, with a r^2^ = 0.34 (p < 0.05) (Fig. S4D). This relation was highly dependent upon the P12 2012 sample (1.7% (w w^−1^ of dry sediment)). No correlation was found with the total-carbon and –phosphate values. Nevertheless, HF183 and *int3* concentration distribution patterns appeared related to the NGS *rrs* genetic structures. Significant correlations were observed between HF183 (indicator of the presence of human fecal matter) concentrations and NMDS ordinations. These relations were in line with those inferred from the total-N contents of the deposits; P12 samples were always harboring this marker and showed the highest values (Table S10). P12 thus concentrated significantly bacteria of fecal origin. Regarding *int3*, their concentrations were significantly correlated to the *rrs* NMDS ordinations (r^2^ = 0.25, p < 0.05) but were also found to significantly explain the *rrs* dataset through RDA. Higher *int3* relative concentrations were related to the 2013/2014 datasets (more recent deposits) (e. g. Fig. S5). This relation between *int3* and the DB bacteriome will require further investigations. The *int3* functional roles remain to be defined. Their high occurrence suggests a key role in bacterial fitness, and likely the degradation or resistance towards chemical pollutants. No statistical relation was found between the *rrs* genetic structures and the hydrological performance indicators or meteorological variables such as preceding dry periods prior sampling that were monitored.

### Inferred bacterial taxa associated with significant changes in the chemical and qPCR datasets

**Figure 6:**
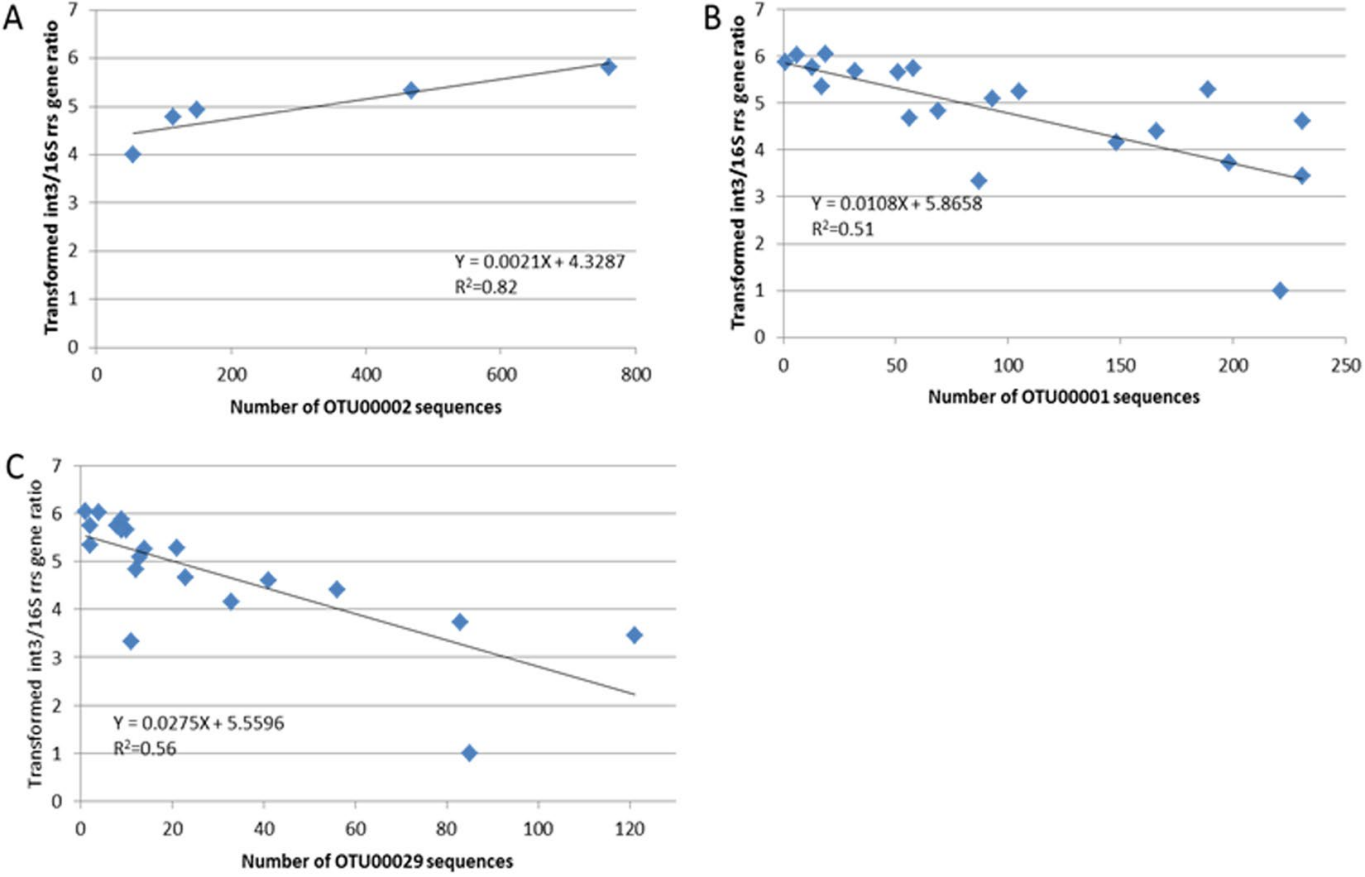
Linear regression analyses showing the relations between *int3* relative concentrations per sample and the number of reads of a particular 16 S rRNA gene OTU. (**A**) *int3* ratios against OTU00002 reads at P12 (decantation pit). (**B**) *int3* ratios against OTU00001 reads for all sampling sites except P12. (**C**) *int3* ratios against OTU00029 reads for all sites except P12. See Fig. 1 for a positioning of the sampling sites. R^2^ is indicated in each panel.

The NMDS and RDA analyses identified patterns in the global *rrs* genetic structures of the investigated bacteriomes. In order to detect OTUs following these patterns, co-occurrence tests were performed between the explanatory variables and the number of reads per OTU. Only OTUs showing more than 15 reads were used. Poorly abundant OTUs were not expected to give significant results while analyzing their distribution over 29 independent datasets. Two sets of analyses were performed: (1) limited to the P12 samples, and (2) all samples except P12. A significant correlation between [*int3*] and the number of reads of OTU00002 when using an OTU contingency table limited to P12 samples (r = 0.97, p < 0.001) (Fig. 6A) was detected. OTU00002 was found related to *Flavobacterium*. However, there is no record of class 3 integrons among *Flavobacterium*. This integron was first found in *Serratia marcescens* TN9106 (isolated from a patient with a urinary tract infection), and has been observed only in Proteobacteria such as *E. coli, Pseudomonas aeruginosa* and *Klebsiella pneumoniae* ^18^. It was associated with gene cassettes encoding beta-lactamases especially *bla*_GES-1_. Interestingly, *Flavobacterium* species showed high level of antibiotic resistances, and were considered a reservoir of antibiotic resistance genes. A direct link with integron 3 elements could thus explain this association but will require further investigations. *Flavobacterium* was mainly found in soil and water systems^18,38^.

Regarding the analyses done on the OTU contingency table limited to the P1, P2, P4 and P7 sites (all dates) (Fig. 6B,C), a few strong correlations could also be recorded. [*int3*] were found to be negatively correlated with 2 OTU distribution patterns i.e. OTU00001 and OTU00029, with r = 0.62 and 0.71, respectively (p < 0.001). OTU00001 was allocated to an unknown genus from the KCM-B-112 family in the Acidithiobacillales (Gamma- proteobacteria). Interestingly, this OTU was first found in a soil near a non-ferrous metal smelting factory named KCM (accession number: AJ581597). OTU00029 was affiliated to *Bradyrhizobium*. This genus is found in soil and water, and was found to be highly resistant to MTE such as zinc, molybdenum or lead^39^. A significant correlation (rho = 0.92, p < 0.001) between OTU00029 sequence numbers and Zn concentrations was observed.

### Concluding remarks

**Figure 7:**
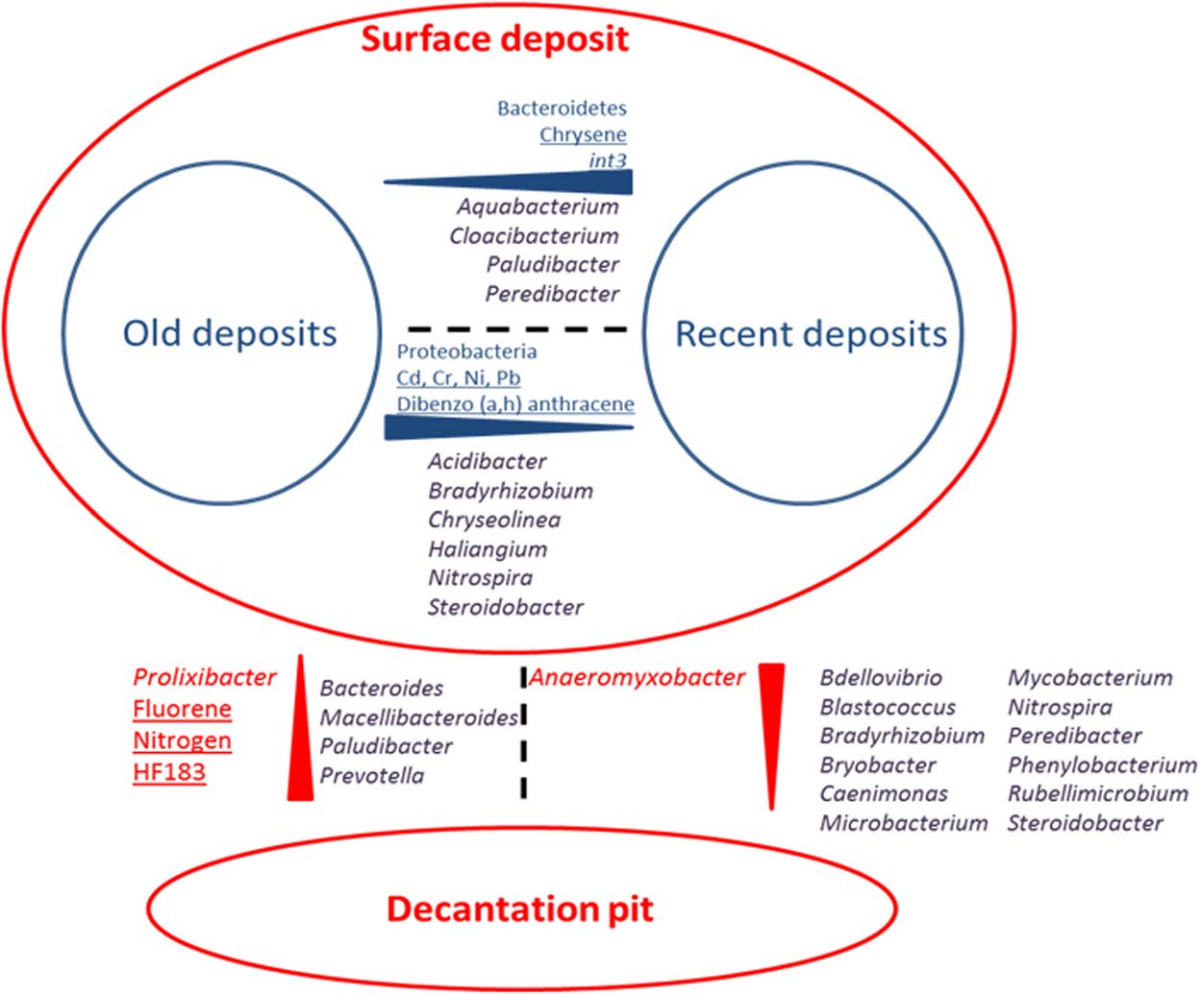
Summary of the relations observed between 16 S rRNA gene sequence inferred bacteriome genetic structures recorded from urban deposits and the monitored explanatory variables. Red highlight differences between the decantation pit and the surface samples of the investigated detention basin, and blue, those observed between old and recent deposits. Names in blue and red are those of variables that showed patterns supported by significant statistical tests. Names in purple highlight other bacterial groups distribution biases that were observed. Most significant Eexplanatory variables are underlined. Red and blue triangles match the observed distribution patterns with their largest portion matching the highest concentrations or number of reads for a particular bacterial group.

Urban ecosystems are divided into patches of variable industrial, residential, recreational, and commercial activities. These activities generate surface deposits made, among others, of urban soils, fecal matter, pyrogenic and petrogenic contaminants. Chemical and microbial contents of these deposits could thus be indicative of their origin and ecotoxicity. Soil particles and rain appeared major sources of the bacterial taxa recovered among recent urban deposits investigated in this work. In fact, *rrs* reads of the genus *Aquabacterium*, which was previously related to rainfall events^40^, were recorded in high numbers among recent surface deposits (Fig. 7). Reads from *Flavobacterium* gave the highest scores for most samples (Fig. 4), and this genus had been previously reported in sediments and soils. Allocation of the *rrs* reads at the genus scale further revealed partitions in the datasets, and these were found to match accumulation time periods of the deposits in the detention basin. This suggested an evolution of urban bacteriomes toward a more specialized structure better adapted for the harsher environmental constraints (higher MTE and dibenzo (a,h) anthracene, see Fig. 7) that were building-up in the deposits. Reads allocated to two genera of extremophiles, *Acidibacter* (reduction of Fe^41^) and *Haliangium* (halophilic genus^42^), were found higher in the older more mature surface deposits of the DB than the recent ones (Fig. 7). General *rrs* genetic structures were also found to be well-differentiated between deposits recovered from the surface (recently or not) of the DB, and those at the bottom of a decantation pit. In fact, *rrs* reads from the investigated decantation pit were found to be enriched in bacterial taxa from the intestinal tract of warm blooded animals i.e. *Bacteroides* and *Prevotella*. This enrichment was related to high concentrations of MST qPCR markers from human *Bacteroides* (HF183) and of *rrs* reads from genera known to be at least facultative anaerobes^43–45^ (Fig. 7). These data thus strongly suggest that these bacteria can live and persist in other biotopes, and outside their native hosts. This very well illustrates the major shifts in bacterial communities that can be driven by man through its management practices. However, these changes were not related to significant enrichments of bacterial genera that could represent significant health hazards. Only reads from the *Acinetobacter*, *Mycobacterium* and *Pseudomonas* genera, which can contain pathogens, were repeatedly recorded in the urban deposits, but no particular segregation of their reads according to the tested explanatory variables could be detected. Other experimental designs will be required to better address issues concerning the ecology of these potentially hazardous taxa. Investigations over the watershed will also be required to highlight human activities involved in the emission processes of some of the observed urban bacterial taxa.

## Materials and Methods

### Experimental site and sampling campaigns

The Chassieu urban industrial catchment (named Mi-Plaine) and its connected DjR detention basin have been described in a previous study^1^. Investigations of variability in the distribution of chemicals and bacteria (inferred from NGS *rrs* and quantitative PCR analyses described below) among urban deposits was addressed by using a nested sampling design. Such a sampling matrix increases the likelihood of identifying key variables (Table S2) in a dataset. These sampling schemes are based on the notion that a population (response variables) can be divided into classes at two or more categorical levels in a hierarchy^46^. The population can thus be sampled with such a scheme to estimate the variance at each level. An observation will embody variation related to each category. This sampling matrix could produce six independent replicates (samples from all dates, n = 6) per sampling point (Table S1), and five replicates (all sampling points in the DB, n = 5) per date (Table S3). Other categories were also considered in this sampling scheme such as maturity of the deposits (old against recent), or their recovery from a decantation pit. These latter categories implied the use of more than six replicates. Five sampling points in the DB were defined according to the hydrodynamic of urban deposits. Sampling points were named P0, P1, P2, P4, P7 and P12, the last one being a decantation pit (Fig. 1). P0 was located outside but close to the edge of the DB and was used as a control. Series of samplings were performed according to time from the last removal of deposits by the manager of the site. Samplings were performed considering global cleanup of the settled deposits among the DB in February 2006 and in April 2013. This allowed analysis of deposits which had been accumulating over a 6 month-period up to about 6 years. Sampling periods were as follow: November 2010 (54-month accumulation period), May 2012 (71-month accumulation period), October 2013 (6 month accumulations), and February (10 month accumulations), April (12.5 month accumulations) and July 2014 (15 month accumulations). During the study period, no major change in the activities and sources of pollutants over the catchment were recorded. The datasets were named according to the sampling year and month, as needed. Characteristics of the campaigns are summarized in Table S1.

### Chemical analyses and DNA extractions

The quartering method was used to generate the most representative samples from urban deposits, according to Gy^47^. The quartering method requires that a “sampling” square area (50 × 50 cm) is defined, and then divided by four. All urban deposits were then taken out from two of these quarters. These quarters were mixed and divided again into a square with a homogeneous thickness. Quarters were made from this new sampling square, and two of these were sampled. This step was repeated three times. Samples taken from the last mix of quarters were used for the chemical and bacterial analyses. Thickness of the deposits at the sampling sites was never over 20 cm making possible the use of this quartering approach. This approach had been previously validated by Sébastian *et al*.^1^. For the control point (P0) at the edge but outside the detention basin, the soil horizon was recovered down to 10 cm over a 50 × 50 cm area. The quartering approach was then used to obtain a representative sample. These samples were put in glass bottles for organic compounds analyses, and in plastic bottles for mineral and global chemical analyses as indicated in Sebastian *et al*.^1^. For the 2012 samples, chemical datasets were taken from Sebastian *et al*.^1^. Deposits were kept at 4 °C for less than 24 h prior analysis. The list of compounds monitored is given in Table S2. Metallic Trace Elements (MTE) were analyzed according to NF EN ISO 11885, 16 Polycyclic Aromatic Hydrocarbons (PAH) according to NF × 33–012, particle size (using a Malvern Mastersizer 2000 granulometer, Orsay cedex, France) according to NF ISO 13320, their bulk density according to ISO NF × 31–501, their dry matter according to NF EN ISO 11465, and volatile matter according to NF EN 12879. Three independent DNA extracts per sampled urban deposits (or soil for P0) were performed using the FastDNA SPIN® Kit (For Soil) (BIO 101, Inc., Carlsbad, France). About 600 mg of deposits were used per DNA extraction. DNA was measured using a nanodrop UV-Vis Spectrophotometer, and kept at −80 °C until further manipulations.

### Monitoring of integrons and human fecal markers by qPCR

PCR amplification was performed using a Bio-Rad CFX96 real-time PCR instrument with Bio-Rad CFX Manager software, version 3.0 (Marnes-la-Coquette, France). The primers and probes used in the present study are summarized in Table S12. Primer3 software version 0.4.0.0 (http://frodo.wi.mit.edu/primer3/) was used for the design of novel primers targeting CDS (coding sequence) encoding integrases of class 2 or 3. Primers for the CDS integrase of class 1 were taken from the literature. The specificity of each oligonucleotide was checked with the BLAST program. All primers were synthesized by Invitrogen (Paris, France) and the probe was synthetized by Eurogentech (Liège, Belgium). The mix reaction was performed with the Brilliant II SYBR green low ROX qPCR master mix for SYBR Green qPCR and the Brilliant II SYBR Green low ROX qPCR master mix (Agilent, Vénissieux, France) for the TaqMan qPCR. Two microliters of template DNA (pooled DNA extracts for each sampling point) was added, and ultrapure water was used to reach a final volume of 20 μl. Negative controls without template DNA were run in triplicate. Each reaction was run in triplicate with the following cycle conditions: 1 cycle at 95 °C for 10 min followed by 40 cycles of 95 °C for 15 sec and annealing temperature for 30 sec. For SYBR Green qPCR, a melting curve step was added in order to check the purity of the PCR products. This step consisted of a ramp temperature from 65° to 95 °C, with an increment of 0.5 °C and holding for 5 sec for each step.

Targeted DNA fragments for Bacteroidales MST markers were cloned into the pGEM-T easy plasmid according to the manufacturer (Promega, Charbonnières-les-Bains, France) and transformed into *Escherichia coli* JM110 strain (Stratagene, Massy, France). The *int1*, *int2* and *int3* integrase CDS were subcloned in *E. coli* by Marti *et al*.^48^. These constructs were kindly provided by Dr. E. Topp (AAFC London, Ontario, Canada). Plasmids were extracted using the Qiagen plasmid midi kit (Qiagen, Courtaboeuf, France). The plasmids were linearized by *Not*I (New England BioLabs, Evry, France) and purified with the Qiagen QIAquick PCR purification kit. Plasmid copy numbers were estimated by NanoDrop ND1000 spectrophotometry (Thermo Fisher Scientific, Villebon-sur-Yvette, France). Standard curves consisted of 10-fold serial dilutions of a known concentration of plasmid solution for each marker. The identities of the quantified gene targets were verified through analysis of the hybridization datasets of the internal probe when the TaqMan chemistry was used or of the melting curves when using SYBR green staining. Presence of inhibitors was checked by spiking known amount of plasmid harboring *int2* with 10 times dilution of sample DNA extracts (10^7^ copies of plasmid per μl of DNA diluted solution). Reactions were duplicated. Number of cycles needed to have a significant signal was compared with wells where only plasmid harboring *int2* was added to the qPCR mix. When a higher number of cycles was needed to observe a signal, a 5 or 10 times dilution was further performed, and another test was realized in order to confirm the absence of PCR inhibitions.

### DNA sequencing of PCR products and Mothur processing

The V5 and V6 segments of 16 S rRNA genes (*rrs*) were targeted in these investigations. For the “2010/2012 dataset”, PCR products were generated from urban deposits (inside the detention basin) or soil (recovered on the edge of the basin as a control point) DNA extracts according to De Filippo *et al*.^49^. These products were sequenced by DNA Vision (Gosselies, Belgium) on a 454 Life Sciences Genome Sequencer FLX instrument (Roche, Meylan, France) (using the following primers: forward 5′ AGGATTAGATACCCTGGTA 3′ and reverse 5′ CAACACGAGCTGACGAC 3′) using Titanium chemistry. For the “2013/2014 dataset”, the *rrs* V5-V6 DNA sequences were obtained using the MiSeq sequencing technology (Illumina, Paris, France) (using the following primers: forward 5′ AMCMGGATTAGATACCCKG and reverse 5′ CRTCCMCACCTTCCTC 3′). Illumina sequencings were performed at Molecular Laboratories (Molecular lab, Shallowater, Texas, USA). In silico analysis of the phylogenetic coverage of the two primer sets against the Silva database was tested. No significant difference in coverage between these primer pairs was found (Wilcoxon test: p = 0.34). The *rrs* DNA libraries were treated using standard operating procedures available in the mothur suite^50^. These allowed removing chimeric sequences, primers, barcodes, and limit the dataset to sequences of a minimum length of 200 bp (average length = 263 bp for the 2010/2012 dataset, and 375 bp for the 2013/14 one). The Silva *rrs* bacterial database (v123; www.arb-silva.de) was used for the alignment step. The split.abund step was used to remove groups containing only 1 sequence after pre-clusterization. This step decreased the amount of unique sequences by 93% and the total amount of sequences by 41%, and decreased drastically time processing of the datasets and file sizes. The 2010/2012 and 2013/14 libraries were merged and aligned before building up the distance matrix and performing the clustering. The number of sequences was normalized between the samples and was set to 8727 sequences. The average sequence length of the merged 2010/2012–2013/14 datasets was 347 pb. A representative sequence per OTU was used in order to make the phylogenetic taxonomic allocations using the Silva bacterial 16 S rRNA gene sequence database (v123) using a cutoff set at the level of identical reads in order to minimize affiliation errors due to short length sequences. One sample from the 2010/2012 dataset (P2 2012) was re-sequenced by the Illumina technology. The OTU contingency table of these P2 2012 datasets were compared, and showed similar distribution patterns of the reads by AMOVA (p = 0.626). Computing of Bray-Curtis dissimilarities between these P2 datasets, and between these datasets and those from other samples (see below) gave similar values (Kruskal-Wallis, p > 0.6). These two datasets were thus combined and analyzed simultaneously. OTU sequences were submitted to the EMBL database under the project accession number ERP023590

### Bioinformatics and statistical analyses

#### general features

Alpha diversity indices (Shannon) and beta diversity (Bray-Curtis) analyses were computed using the Mothur suite^50^. Contingency tables encompassing OTU affiliations were imported into the Explicet software^51^ in order to generate heatmaps and pie charts.

#### OTU partition analyses

All statistical analyses were made from OTU contingency tables except when mentioned otherwise. In order to assess the impact of sampling locations and years on the datasets, (1) UniFrac weighted and unweighted tests were performed on Bray Curtis matrices, and (2) non parametric AMOVA tests were performed (number of iterations = 1000 and alpha parameter = 0.05); both were done as described in the MiSeq standard operating procedures from mothur. R software^52^ was used to perform: (1) non-metric multi-dimensional scaling (with a square root transformation followed by a double Wisconsin standardization), (2) redundancy analyses with Hellinger transformation for OTU contingency tables and Log_10_ transformation for metadata using the decostand command from the Vegan package as described in Gobet *et al*.^53^, (3) Spearman rank correlation tests, and (4) principal component analyses. Akaike’s Information Criterion (AIC) was used to highlight the variables explaining constraint ordination inertia using permutation tests. Permutation tests (n = 1000) were used to test significance of variables and OTU associations with a p-value threshold set at 0.05 as described in the Vegan R package manual^54^.

#### Correlation tests

The CoNet application implemented in the Cytoscape software was used to highlight correlations between bacterial OTUs, and physical and chemical parameters^55,56^. Correlations were computed using Pearson correlation tests (r > 0.6). Strength of the correlations were tested by 1,000 bootstrap and the Bonferroni multi-test correction (set at p < 0.05).

## Electronic supplementary material

Supplementary materials
