# Supplementary material for: Bacteriome genetic structures of urban deposits are indicative of their origin and impacted by chemical pollutants
Source: Sci Rep. 2017 Oct 16;7:13219. doi: 10.1038/s41598-017-13594-8 (PMC5643393; doi:10.1038/s41598-017-13594-8)
Supplement: Supplementary file 1 — Supplementary materials [file 41598_2017_13594_MOESM1_ESM.doc]

**Bacteriome genetic structures of urban deposits are indicative of their origin and impacted by chemical pollutants**

Romain Marti^1^, Céline Bécouze-Lareure^2^, Sébastien Ribun^1^, Laurence Marjolet^1^, Claire Bernardin -Souibgui^1^, Jean-Baptiste Aubin^2^, Gislain Lipeme Kouyi^2^, Laure Wiest^3^, Didier Blaha^1^, Benoit Cournoyer^1*^

^1^Research Group on “Bacterial Opportunistic Pathogens and Environment”, UMR CNRS5557, INRA1418 Ecologie Microbienne, Université Lyon 1, VetAgro Sup, Marcy L’Etoile, France, ^2^DEEP, INSA Lyon, Villeurbanne, France, ^3^Institut des Sciences Analytiques UMR5280, Villeurbanne, France

*Corresponding author: benoit.cournoyer@vetagro-sup.fr


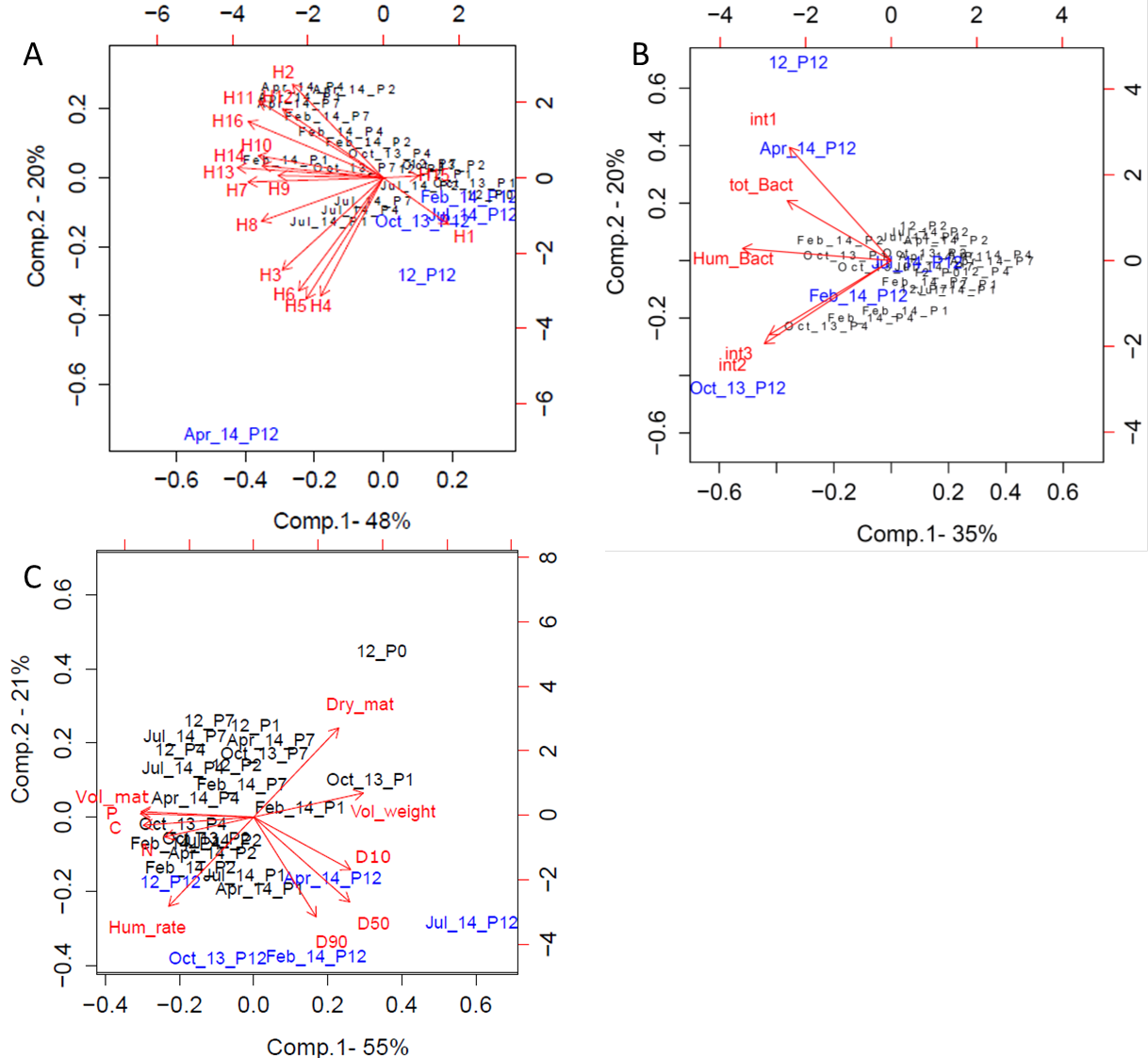


Figure S1. Principal component analyses (PCA) performed on the physical, chemical and molecular datasets. Relation between sampling date and point, and (A) PAH concentrations, (B) qPCR bacterial marker concentrations, and (C) general features of the sediments and campaigns. See Table S1 & S2 for the full names of the tested parameters, and Fig. 1 for position (abbreviated P) of the samples named according to the sampling month (month_year) and/or year. Red arrows indicate the measured parameters.


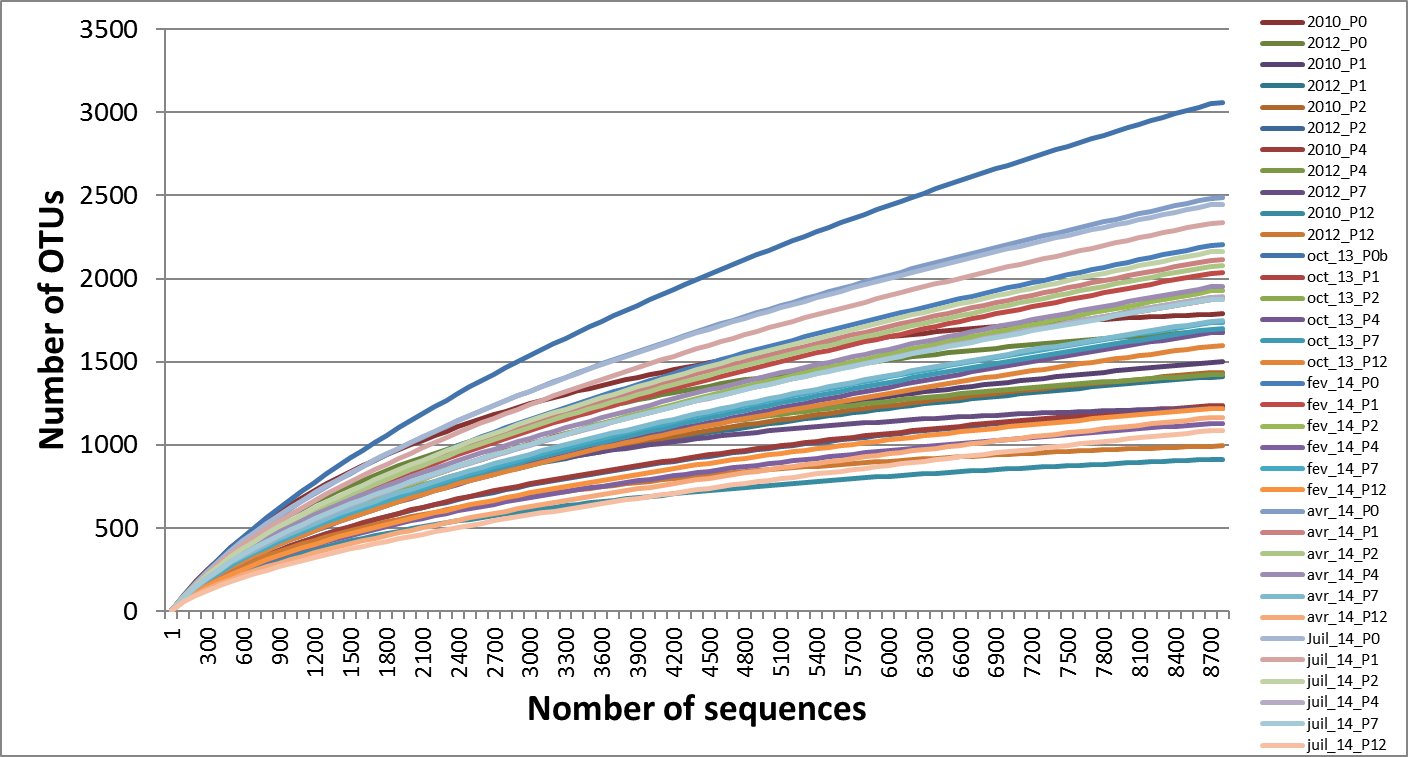


Oct 13 P0

Oct 14 P0

July14 P0

July14 P1

Figure S2. Rarefaction curves illustrating the relation between the number of 16S rRNA gene sequences reads and OTU per sample. See Fig. 1 for position of the samples.


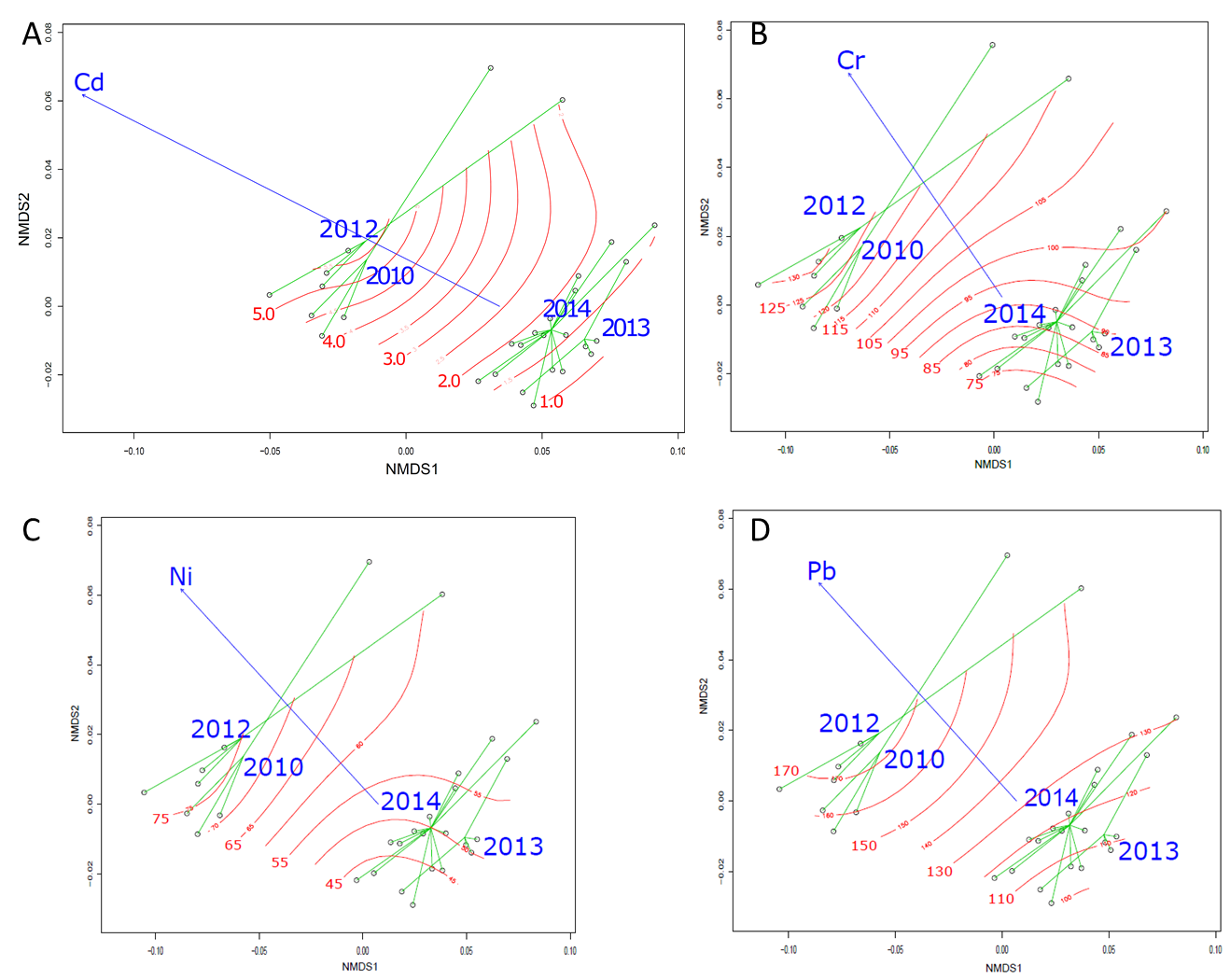
Figure S3. Correlations between TEM concentrations and NMDS ordinations of the number of 16S rDNA reads per OTU recovered from the sampled sediments. Small opened circles represent the samples. Each blue arrow per panel represents the relation with a particular ETM: (A) Cd, (B) Cr, (C) Ni and (D) Pb. Direction of the arrow indicates a significant correlation. Red lines indicate the estimated concentrations according to the dataset (in μg g^-1^ of dry sediment) (Table S9). Green lines are grouping points according to the sampling year. See Fig. 1 for position of the samples.


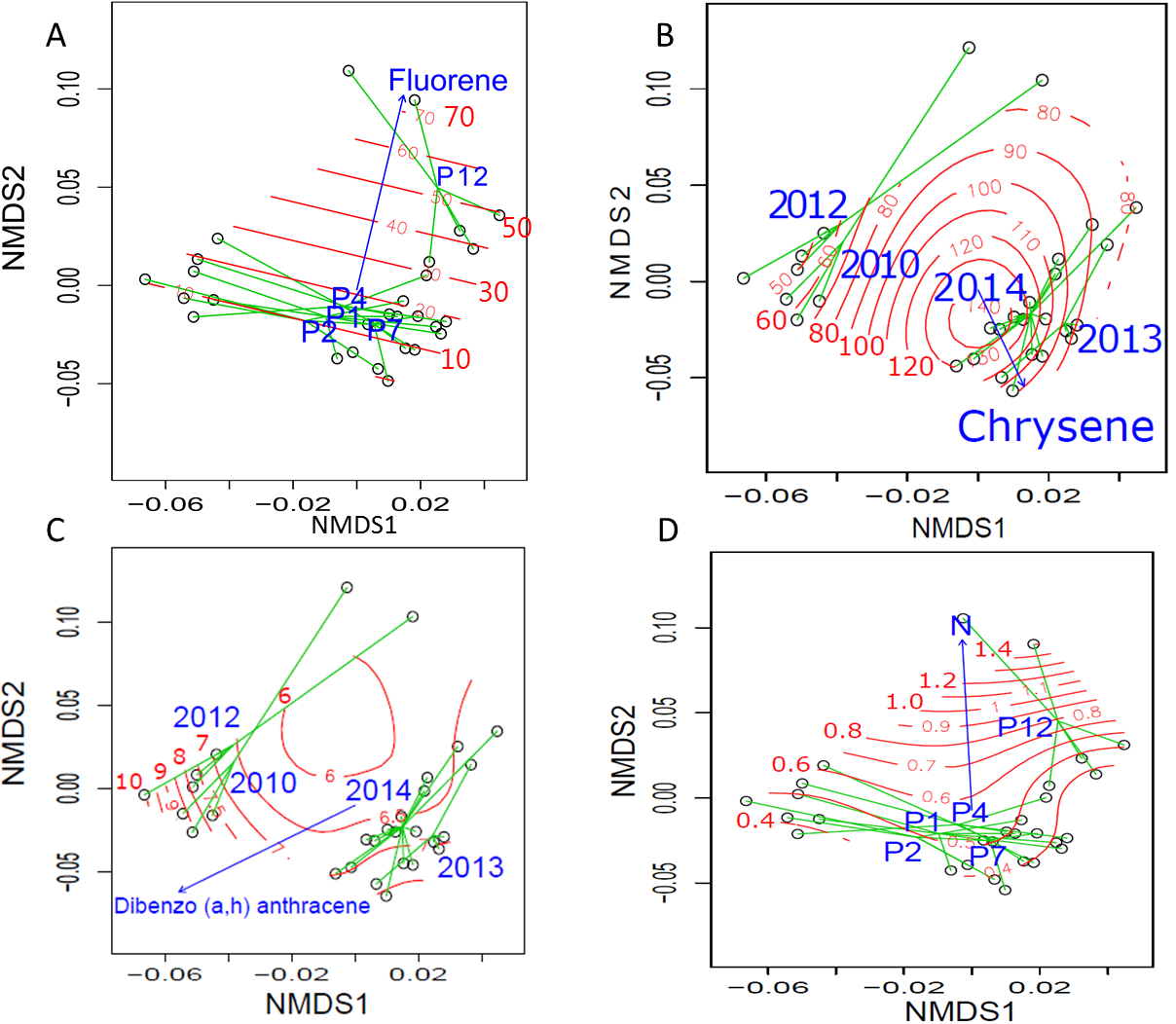


Figure S4. Correlations between PAH concentrations, total N-content, and NMDS ordinations of the number of 16S rDNA reads per OTU recovered from the sampled sediments. Small opened circles represent the samples. Each blue arrow per panel represents the relation with (A) Fluorene, (B) Chrysene, (C) Dibenzo (a,h) anthracene, and (D) with total nitrogen content of each sampled sediment. Direction of the arrow indicates a significant correlation. Red lines indicate the estimated concentrations according to the dataset (in μg g^-1^ of dry sediment) or the nitrogen ratio (W W^-1^ of dry matter). Green lines are grouping points according to the sampling year or site. See Fig. 1 for position of the samples.


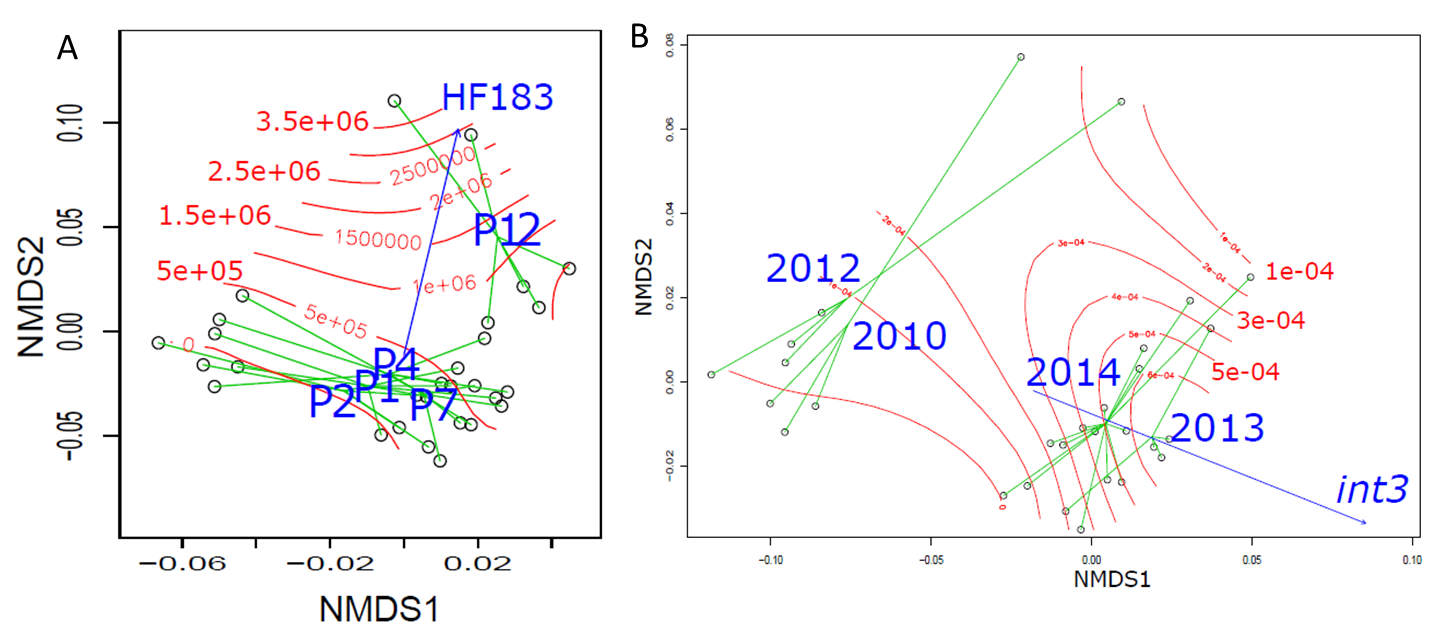


Figure S5. Correlations between concentrations of bacterial markers (qPCR datasets), and NMDS ordinations of the number of 16S rDNA reads per OTU recovered from the sampled sediments. Small opened circles represent the samples. Each blue arrow per panel represents the relation with (A) HF183 and (B) *int3* relative ratios expressed per number of 16S rDNA qPCR products per sampled sediment. Direction of the arrow indicates a significant correlation. Red lines indicate the estimated ratios. Green lines are grouping points according to the sampling year or site. See Fig. 1 for position of the samples.

| Table S1. Main features of the field sampling campaigns | | | |  | | |  | |  |  | | |  | |  | |
| --- | --- | --- | --- | --- | --- | --- | --- | --- | --- | --- | --- | --- | --- | --- | --- | --- |
| Name | Sampling date | Conditions | | Period without  maintenance  (months) | | | Sampling  season | | ADP^a^ (d) | Rd^b^ (h) | | | Ht^c^ (mm) | | Imax_5_^d^ (mm/h) | |
| 2010 | 29 November 2010 | | 28 months before cleaning | | 54 | Winter | | 3.1 | | | 1.5 | 2.9 | | 5.9 | |  |
| 2012 | 02 May 2012 | | 11 months before cleaning | | 71 | Spring | | 2.0 | | | 7.7 | 33.3 | | 266.3 | |  |
| Oct_2013 | 02 October 2013 | | 6 months after cleaning | | 6 | Autumn | | 2.2 | | | 26.7 | 16.0 | | 11.6 | |  |
| Feb_2014 | 03 February 2014 | | 10 months after cleaning | | 10 | Winter | | 1.1 | | | 17.6 | 11.8 | | 3.8 | |  |
| Apr_2014 | 24 April 2014 | | 12.5 months after cleaning | | 12.5 | Spring | | 1.4 | | | 5.4 | 2.5 | | 5.5 | |  |
| Jul_2014 | 04 July 2014 | | 14.8 months after cleaning^e^ | | 14.8 | Summer | | 1.4 | | | 27.3 | 11.4 | | 9.8 | |  |
| ^a^ ADP: antecedent dry period before sampling; ^b^ Rd: cumulative rainfall duration – over one week prior sampling; ^c^ Ht: total rainfall - over one week prior sampling; ^d^ Imax_5_ maximum rainfall intensity at a 5-min time step - over one week prior sampling. ^e^ The decantation pit (P12 point) was emptied between April and July 2014 sampling campaigns. | | | | | | | | | | | | | | | | |

Table S2. Bacterial markers, chemicals and physical parameters investigated in this study

| Molecular markers | Integrase CDS from class 1, 2, and 3 integrons, bacterial 16S rRNA gene segments, HF183 16S rDNA marker for a human specific Bacteroidales species, and a 16S rDNA segment distributed among all Bacteroidales |
| --- | --- |
| Physical parameters | water content, volatile organic matter (VM), particle size distribution patterns (D10, D50, D90), bulk density |
| MTE | Cadmium, Lead, Copper, Zinc, Chromium and Nickel |
| Nutrients | Total Nitrogen (N), Carbon (C) and Phosphorus (P) concentrations |
| PAHs | Naphtalene (H1), Acenaphthylene (H2), Acenaphtene (H3), Fluorene (H4), Phenanthrene (H5), Anthracene (H6), Fluoranthene (H7), Pyrene (H8), Benzo(a)anthracene (H9), Chrysene (H10), Benzo(b)fluoranthene (H11), Benzo(k)fluoranthene (H12), benzo(a)pyrene (H13), Indeno(1,2,3-cd)pyrene (H14), Dibenzo(a,h)anthracene (H15), Benzo(g,h,i)perylene (H16) |

| Table S3. Water content of deposits of the DjR detention basin according to sampling time. | | | | | |  | | |
| --- | --- | --- | --- | --- | --- | --- | --- | --- |
| Sample site | 2012 | Oct 2013 | Feb 2014 | Apr 2014 | Jul 2014 |  | | |
| P1 | 37.6% ± 2.0% | 30.5% ± 2.8% | 41.0% ± 2.4% | 50.5% ± 1.6% | 48.1% ± 1.7% |  | | |
| P2 | 44.4% ± 1.8% | 51.3% ± 2.0% | 55.4% ± 1.8% | 47.8% ± 1.7% | 44.4% ± 1.8% |  | | |
| P4 | 41.1% ± 2.0% | 54.5% ± 1.8% | 59.2% ± 1.6% | 63.4% ± 2.5% | 35.1% ± 2.1% |  | | |
| P7 | 35.6% ± 2.1% | 39.3% ± 2.4% | 46.5% ± 2.1% | 31.5% ± 2.2% | 33.4% ± 2.2% |  | | |
| P12 | 60.1% ± 1.3% | 70.3% ± 1.0% | 62.6% ± 1.5% | 50.5% ± 1.6% | 19.7% ± 2.6% |  | | |
| Control point P0 had values ranging from 1 to 16%. See Fig. 1 for position of the sampling sites and Table S1 for the main features of the sampling campaigns. | | | | | |  |  |  |

| Table S4. Distribution of particle sizes (µm) per sampling site and campaign. | | | | | |
| --- | --- | --- | --- | --- | --- |
| Sample site | 2012 | Oct 2013 | Feb 2014 | Apr 2014 | Jul 2014 |
| D10 |  |  |  |  |  |
| P1 | 6.1 ± 23.6% | 30.7 ± 13.0% | 25.2 ± 18.8% | 24.3 ± 10.8% | 18.2 ± 14.3% |
| P2 | 8.4 ± 5.9% | 9.7 ± 13.0% | 13.9 ± 1.9% | 16.8 ± 1.5% | 12.3 ± 14.8% |
| P4 | 6.6 ± 8.6% | 7.1 ± 3.0% | 10.2 ± 0.9% | 10.7 ± 1.7% | 11.0 ± 4.5% |
| P7 | 5.5 ± 6.3% | 8.4 ± 22% | 12.0 ± 1.5% | 14.5 ± 4.6% | 12.5 ± 4.6% |
| P12 | 8.4 ± 30.8% | 15.8 ± 7.0% | 34.1 ± 14.9% | 20.5 ± 4.6% | 192.5 ± 6.7% |
| D50 |  |  |  |  |  |
| P1 | 37.1 ± 22.2% | 193.6 ± 6.0% | 134.9 ± 3.8% | 165.4 ± 9.2% | 141.1 ± 13.6% |
| P2 | 50.4 ± 4.0% | 67.9 ± 11.0% | 88.2 ± 2.8% | 108.2 ± 2.8% | 89.3 ± 19.0% |
| P4 | 33.9 ± 4.8% | 45.2 ± 2.0% | 54.2 ± 4.0% | 58.9 ± 2.0% | 60.8 ± 9.5% |
| P7 | 33.9 ± 3.5% | 48.2 ± 11.0% | 57.8 ± 0.8% | 69.1 ± 7.6% | 71.3 ± 2.3% |
| P12 | 61.6 ± 3.5% | 172.3 ± 17.0% | 318.0 ± 7.6% | 245.2 ± 4.6% | 534.6 ± 0.4% |
| D90 |  |  |  |  |  |
| P1 | 175.7 ± 43.9% | 492.0 ± 11.0% | 451.5 ± 4.1% | 790.3 ± 13.9% | 799.3 ± 13.9% |
| P2 | 243.6 ± 10.3% | 478.4 ± 12.0% | 512.7 ± 10.2% | 651.3 ± 12.8% | 651.3 ± 33.2% |
| P4 | 102.7 ± 4.9% | 225.8 ± 6.0% | 212.5 ± 24.3% | 197.3 ± 1.6% | 305.3 ± 41.1% |
| P7 | 156.9 ± 13.4% | 171.9 ± 13.0% | 182.6 ± 4.5% | 348.2 ± 30.3% | 307.6 ± 1.5% |
| P12 | 335.0 ± 34.7% | 733.17 ± 10.0% | 748.4 ± 5.0% | 682.2 ± 6.2% | 1094.6 ± 0.1% |
| Control point P0: D10 between 3.6 µm and 22.9 µm. D50 between 33.9 µm and 263.6 µm. D90 between 289.8 µm and 735.9 µm. See Fig. 1 for position of the sampling sites and Table S1 for the main features of the sampling campaigns. | | | | | |

| Table S5. Bulk density of deposits of the DjR detention basin (kg.m^-3^) | | | | | |
| --- | --- | --- | --- | --- | --- |
| Sample site | 2012 | Oct 2013 | Feb 2014 | Apr 2014 | Jul 2014 |
| P1 | 2475.0 ± 2.6% | 2516.8 ± 0.5% | 2461.0 ± 18.2% | 2455.0 ± 18.6% | 2289.7 ± 11.8% |
| P2 | 2350.0 ± 1.1% | 2244.3 ± 0.9% | 2264.0 ± 0.7% | 2289.0 ± 3.4% | 2225.4 ± 15.0% |
| P4 | 2460.0 ± 1.1% | 2194.5 ± 0.6% | 2242.0 ± 1.1% | 2170.0 ± 8.0% | 2325.5 ± 11.9% |
| P7 | 2393.0 ± 0.6% | 2304.5 ± 2.5% | 2377.0 ± 0.1% | 2562.0 ± 21.0% | 2311.4 ± 10.0% |
| P12 | 2303.0 ± 3.5% | 2320.8 ± 0.4% | 2445.0 ± 0.3% | 2575.0 ± 16.2% | 2636.4 ± 34.1% |
| Control point P0: 2527 kg.m^-3^ and 2653 kg.m^-3^. See Fig. 1 for position of the sampling sites and Table S1 for the main features of the sampling campaigns | | | | | |

| Table S6. Relative content in volatile organic matter of the deposits of the DjR detention basin (% of dry matter) | | | | | |
| --- | --- | --- | --- | --- | --- |
| Sample site | 2012 | Oct 2013 | Feb 2014 | Apr 2014 | Jul 2014 |
| P1 | 21.2% | 5.9% | 10.0% | 25.0% | 23.0% |
| P2 | 17.7% | 21.3% | 21.7% | 24.5% | 18.7% |
| P4 | 25.5% | 22.5% | 24.2% | 22.9% | 20.9% |
| P7 | 25.6% | 17.1% | 17.0% | 21.0% | 21.1% |
| P12 | 17.9% | 19.0% | 15.0% | 9.8% | 3.1% |
| Control point P0: from 2.3% to 6%. See Fig. 1 for position of the sampling sites and Table S1 for the main features of the sampling campaigns | | | | | |

| Table S7. Relative content in total nitrogen and carbon per g of deposits dry matter from the DjR detention basin, and total phosphorus in µg.g^-1^ of dry sediment | | | | | |
| --- | --- | --- | --- | --- | --- |
| Sample site | 2012 | Oct_2013 | Feb_2014 | Apr_2014 | Jul_2014 |
| N |  |  |  |  |  |
| P1 | 0.4% ± 0.1% | 0.1% ± 0.0% | 0.3% ± 0.1% | 0.5% ± 0.1% | 0.3% ± 0.1% |
| P2 | 0.8% ± 0.2% | 0.4% ± 0.1% | 0.7% ± 0.1% | 0.6% ± 0.1% | 0.5% ± 0.1% |
| P4 | 0.7% ± 0.1% | 0.5% ± 0.1% | 0.6% ± 0.1% | 0.5% ± 0.1% | 0.6% ± 0.1% |
| P7 | 0.5% ± 0.1% | 0.3% ± 0.1% | 0.4% ± 0.1% | 0.4% ± 0.1% | 0.6% ± 0.1% |
| P12 | 1.7% ± 0.3% | 0.6% ± 0.1% | 0.3% ± 0.1% | 0.3% ± 0.1% | 0.1% ± 0.0% |
| C |  |  |  |  |  |
| P1 | 10.3% ± 4.1% | 5.7% ± 2.3% | 10.5% ± 4.2% | 9.2% ± 3.7% | 9.2% ± 3.7% |
| P2 | 10.0% ± 4.0% | 13.1% ± 5.2% | 15.7% ± 6.3% | 13.9% ± 5.6% | 12.6% ± 5.0% |
| P4 | 10.8% ± 4.3% | 15.3% ± 6.1% | 15.5% ± 6.2% | 14.7% ± 5.9% | 14.0% ± 5.6% |
| P7 | 10.0% ± 4.0% | 12.2% ± 4.9% | 11.5% ± 4.6% | 13.6% ± 5.4% | 12.6% ± 5.0% |
| P12 | 9.6% ± 3.8% | 12.0% ± 4.6% | 10.8% ± 4.3% | 6.0% ± 2.4% | 2.7% ± 1.1% |
| P |  |  |  |  |  |
| P1 | 1219.0 ± 272.6 | 553.0 ± 123.7 | 855.0 ± 191.2 | 1125.0 ± 251.6 | 1420.0 ± |
| P2 | 1271.0 ± 284.3 | 1368.0 ± 305.9 | 1311.5 ± 293.3 | 1323.0 ± 295.8 | 1460.0 ± |
| P4 | 1345.0 ± 300.8 | 1234.0 ± 275.9 | 1271.0 ± 272.1 | 1161.0 ± 259.6 | 1330.0 ± |
| P7 | 1223.0 ± 273.5 | 923.0 ± 206.4 | 1066.0 ± 238.4 | 1016.0 ± 238.4 | 1370.0 ± |
| P12 | 1432.0 ± 320.2 | 1096.0 ± 245.1 | 838.0 ± 187.4 | 838.0 ± 187.4 | 531.0 ± |

Control point P0: N, from 0.05% to 0.37% of dry matter; C, from 2.1% to 4.4% of dry matter; P: from 333.0 µg.g^-1^ to 728.0 µg.g^-1^ of dry matter. See Fig. 1 for position of the sampling sites and Table S1 for the main features of the sampling campaigns

Table S8. Concentrations of PAH among deposits of the DjR detention basin (ng g^-1^ of dry deposit)

| Sampling time | Position | H1* | H2 | H3 | H4 | H5 | H6 | H7 | H8 | H9 | H10 | H11 | H12 | H13 | H14 | H15 | H16 |
| --- | --- | --- | --- | --- | --- | --- | --- | --- | --- | --- | --- | --- | --- | --- | --- | --- | --- |
| 2012 | P0 | 36.1 | 1.2 | 0.7 | 2.6 | 18.1 | 1.4 | 27.5 | 19.1 | 3.1 | 15.9 | 5.5 | 8.4 | 2.6 | 8.5 | 8.5 | 8.5 |
|  | P1 | 21.6 | 1.0 | 4.4 | 7.7 | 45.1 | 13.9 | 75.9 | 62.5 | 15.1 | 30.4 | 67.0 | 27.6 | 29.7 | 19.1 | 7.2 | 33.1 |
|  | P2 | 37.8 | 2.7 | 34.0 | 20.3 | 83.8 | 20.4 | 168.4 | 125.1 | 45.3 | 51.2 | 99.9 | 29.1 | 58.5 | 30.6 | 11.0 | 49.0 |
|  | P4 | 27.1 | 1.4 | 14.3 | 12.6 | 52.2 | 16.1 | 109.4 | 86.1 | 23.1 | 36.9 | 97.2 | 34.4 | 41.0 | 34.4 | 7.0 | 51.7 |
|  | P7 | 35.5 | 2.1 | 4.4 | 6.9 | 44.4 | 16.6 | 98.2 | 78.0 | 21.4 | 36.9 | 97.4 | 37.2 | 27.5 | 28.3 | 7.3 | 46.8 |
|  | P12 | 80.2 | 4.4 | 54.0 | 103.4 | 234.0 | 1.0 | 193.6 | 177.0 | 19.1 | 38.1 | 39.6 | 11.9 | 16.3 | 9.2 | 6.2 | 26.4 |
| Oct_2013 | P0 | - | - | - | - | - | - | - | - | - | - | - | - | - | - | - | - |
|  | P1 | 0.9 | 1.1 | 2.1 | 2.4 | 31.0 | 1.2 | 44.0 | 57.3 | 33.9 | 22.8 | 29.9 | 4.9 | 2.3 | 15.1 | 7.6 | 15.1 |
|  | P2 | 0.8 | 0.9 | 1.8 | 2.0 | 27.5 | 3.5 | 45.3 | 79.0 | 63.7 | 27.1 | 72.8 | 16.5 | 27.5 | 6.6 | 6.6 | 37.1 |
|  | P4 | 0.7 | 0.9 | 1.8 | 2.0 | 88.5 | 1.0 | 161.4 | 166.9 | 99.6 | 79.6 | 110.9 | 36.7 | 34.9 | 12.8 | 6.4 | 53.1 |
|  | P7 | 0.8 | 1.0 | 28.4 | 7.3 | 195.0 | 1.1 | 217.8 | 199.4 | 125.0 | 97.7 | 141.3 | 40.0 | 50.0 | 35.6 | 7.1 | 62.1 |
|  | P12 | 2.2 | 0.8 | 45.6 | 34.9 | 238.8 | 0.9 | 200.6 | 161.0 | 2.2 | 58.3 | 59.5 | 20.4 | 17.9 | 11.6 | 5.8 | 11.6 |
| Feb_2014 | P0 | - | - | - | - | - | - | - | - | - | - | - | - | - | - | - | - |
|  | P1 | 0.8 | 9.4 | 92.5 | 58.4 | 100.0 | 24.6 | 276.2 | 192.1 | 104.9 | 171.2 | 161.2 | 64.8 | 88.7 | 68.7 | 7.1 | 96.0 |
|  | P2 | 0.7 | 10.1 | 37.8 | 13.5 | 67.4 | 17.2 | 144.9 | 141.0 | 40.0 | 115.3 | 111.8 | 36.0 | 45.5 | 51.8 | 6.4 | 91.4 |
|  | P4 | 0.7 | 12.5 | 38.7 | 16.8 | 83.1 | 14.9 | 200.6 | 162.7 | 50.0 | 128.2 | 125.1 | 38.4 | 55.0 | 78.2 | 6.2 | 107.5 |
|  | P7 | 0.8 | 14.1 | 25.5 | 7.0 | 105.7 | 17.5 | 253.8 | 181.2 | 59.4 | 141.7 | 142.4 | 44.5 | 61.0 | 71.3 | 6.8 | 109.4 |
|  | P12 | 0.7 | 0.9 | 22.9 | 6.3 | 44.3 | 1.0 | 54.2 | 46.7 | 10.7 | 76.2 | 40.2 | 1.2 | 1.8 | 6.1 | 6.1 | 6.1 |
| Apr_2014 | P0 | - | - | - | - | - | - | - | - | - | - | - | - | - | - | - | - |
|  | P1 | 0.8 | 19.6 | 35.1 | 14.5 | 93.1 | 26.0 | 220.3 | 184.3 | 64.0 | 135.7 | 146.3 | 63.0 | 81.8 | 105.3 | 6.6 | 132.7 |
|  | P2 | 0.8 | 15.6 | 16.6 | 6.9 | 78.5 | 17.5 | 160.1 | 155.3 | 44.2 | 121.6 | 139.1 | 55.3 | 70.0 | 81.5 | 6.7 | 120.7 |
|  | P4 | 0.8 | 16.8 | 24.1 | 7.0 | 82.8 | 17.5 | 212.9 | 215.5 | 61.1 | 144.2 | 173.9 | 55.9 | 74.6 | 97.5 | 6.8 | 143.0 |
|  | P7 | 0.9 | 14.2 | 35.3 | 7.8 | 99.0 | 16.1 | 277.6 | 218.5 | 65.5 | 173.2 | 166.1 | 58.3 | 77.3 | 90.3 | 7.6 | 120.3 |
|  | P12 | 0.8 | 0.9 | 159.7 | 166.4 | 1173.7 | 243.5 | 258.1 | 332.8 | 87.1 | 130.4 | 97.7 | 36.8 | 102.1 | 98.7 | 6.6 | 103.6 |
|  |  |  |  |  |  |  |  |  |  |  |  |  |  |  |  |  |  |
|  |  |  |  |  |  |  |  |  |  |  |  |  |  |  |  |  |  |
|  |  |  |  |  |  |  |  |  |  |  |  |  |  |  |  |  |  |
| Jul_2014 | P0 | 1.1 | 1.3 | 0.8 | 2.9 | 29.9 | 1.5 | 0.6 | 27.1 | 20.6 | 22.6 | 11.2 | 1.8 | 9.6 | 9.5 | 9.5 | 9.5 |
|  | P1 | 0.8 | 0.9 | 79.7 | 2.1 | 95.2 | 63.3 | 247.7 | 295.4 | 112.6 | 173.1 | 80.9 | 1.3 | 54.0 | 113.5 | 6.7 | 57.4 |
|  | P2 | 41.1 | 1.0 | 1.9 | 2.1 | 39.4 | 26.6 | 83.2 | 140.9 | 37.0 | 115.0 | 72.5 | 1.3 | 27.3 | 91.9 | 6.9 | 50.3 |
|  | P4 | 61.5 | 1.0 | 0.6 | 2.3 | 112.8 | 44.6 | 241.8 | 309.3 | 58.0 | 193.5 | 86.5 | 1.4 | 45.4 | 109.5 | 7.4 | 76.1 |
|  | P7 | 45.5 | 1.0 | 2.1 | 2.3 | 83.3 | 47.8 | 201.7 | 306.1 | 54.6 | 169.4 | 76.0 | 1.4 | 35.4 | 109.5 | 7.5 | 63.7 |
|  | P12 | 55.3 | 1.2 | 13.4 | 2.6 | 62.9 | 27.1 | 72.3 | 79.5 | 29.6 | 44.0 | 15.4 | 1.6 | 14.3 | 8.3 | 8.3 | 23.7 |

*H1: Naphtalene, H2: Acenaphthylene, H3: Acenaphtene, H4: Fluorene, H5: Phenanthrene, H6: Anthracene, H7: Fluoranthene, H8: Pyrene, H9: Benzo (a) Anthracene, H10: Chrysene, H11: Benzobfluoranthene, H12: Benzo (k) fluoranthene, H13: Benzo (a) pyrene, H14: Indeno 1,2,3-cdPyrene, H15: Dibenzo (a,h) Anthracene, H16: Benzo (g,h,i) Perylene

| Table S9. Concentrations of metallic trace elements among deposits of the DjR detention basin (μg MTE g^-1^ of dry deposit). | | | | | | |  |
| --- | --- | --- | --- | --- | --- | --- | --- |
| Sample site | 2010 ^a^ | 2012 | Oct_2013 | Feb_2014 | Apr_2014 | Jul_2014 |  |
| Cd^Δ^ |  |  |  |  |  |  |  |
| P1 | 4.7 | 4.1 | 0.7 | - | - | 1.65 |  |
| P2 | 8.3 | 5.5 | 1.7 | - | - | 1.56 |  |
| P4 | -^b^ | 5.6 | 1.7 | - | - | 1.55 |  |
| P7 | 6.8 | 6.6 | 1.5 | - | - | 1.52 |  |
| P12 |  | 1.7 | 1.1 | - | - | 0.61 |  |
| Cr^Δ^ |  |  |  |  |  |  |  |
| P1 | - | 156 | 70 | - | - | 58.2 |  |
| P2 | - | 135 | 129 | - | - | 63.9 |  |
| P4 | - | 137 | 113 | - | - | 53.3 |  |
| P7 | - | 130 | 122 | - | - | 53.7 |  |
| P12 | - | 113 | 111 | - | - | 18.3 |  |
| Cu^Δ§^ |  |  |  |  |  |  |  |
| P1 | 297 | 271 | 138 | - | - | 267 |  |
| P2 | 450 | 312 | 285 | - | - | 250 |  |
| P4 | - | 308 | 275 | - | - | 230 |  |
| P7 | 295 | 279 | 222 | - | - | 218 |  |
| P12 | - | 213 | 229 | - | - | 99.3 |  |
| Ni^Δ^ |  |  |  |  |  |  |  |
| P1 | - | 92 | 42 | - | - | 30.6 |  |
| P2 | - | 85 | 75 | - | - | 32 |  |
| P4 | - | 81 | 72 | - | - | 26.1 |  |
| P7 | - | 82 | 67 | - | - | 26.4 |  |
| P12 | - | 60 | 68 | - | - | 11 |  |
| Pb^Δ§^ |  |  |  |  |  |  |  |
| P1 | 220 | 135 | 67 | - | - | 127 |  |
| P2 | 325 | 164 | 141 | - | - | 129 |  |
| P4 | - | 201 | 138 | - | - | 112 |  |
| P7 | 280 | 231 | 131 | - | - | 118 |  |
| P12 | - | 123 | 115 | - | - | 21.8 |  |
| Zn |  |  |  |  |  |  |  |
| P1 | 1500 | 1558 | 610 | - | - | 1790 |  |
| P2 | 1750 | 1612 | 1673 | - | - | 1790 |  |
| P4 | - | 1663 | 1503 | - | - | 1750 |  |
| P7 | 1570 | 1508 | 1358 | - | - | 1610 |  |
| P12 | - | 1590 | 1380 | - | - | 423 |  |
| Table S9 – complementary annotations: Control point P0: Cd, 0.02 to 0.70 μg g^-1^ of dry matter; Cr, 12.20 to 70.00 μg g^-1^ of dry matter; Cu, 8.92 to 290.00 μg g^-1^ of dry matter; Ni, 8.93 to 42.00 μg g^-1^ of dry matter; Pb, 16.60 to 70.00 μg g^-1^ of dry matter; Zn, 68.3 to 610 μg g^-1^ of dry matter. ^a^ Data obtained from Sébastian et al., 2014; ^b^ Data not available. ^Δ^ Significant difference between 2010/2012 and 2013/2014 datasets (Wilcoxon test, p<0.05). ^§^ Significant difference between P12 datasets and other data (Wilcoxon test p<0.05). See Fig. 1 for position of the sampling sites and Table S1 for the main features of the sampling campaigns. | | | | | | | |

Table S10. qPCR data in Log copy number per g of sediment with their respective standard deviation.

| *int1* | P0 | P1 | P2 | P4 | P7 | P12 |
| --- | --- | --- | --- | --- | --- | --- |
| 2010 | NA | < LOD | < LOD | < LOD | < LOD | < LOD |
| 2012 | < LOD | < LOQ | < LOD | < LOD | < LOQ | 6.4 ± 4.4 |
| Oct_13 | 4.5 ± 3.3 | 6.2 ± 5.3 | 6.2 ± 5.1 | 6.9 ± 5.7 | 6.4 ± 4.7 | 6.8 ± 5.0 |
| Feb_14 | < LOD | 4.2 ± 3.0 | 6.7 ± 4.9 | 5.6 ± 3.6 | 6 ± 4.8 | 5.5 ± 3.8 |
| Apr_14 | < LOD | 6.3 ± 4.5 | 6.4 ± 5.3 | 6.8 ± 5.3 | 4.7 ± 3.5 | 7.1 ± 5.9 |
| Jul_14 | < LOD | < LOD | < LOD | < LOD | < LOD | 4.5 ± 4.4 |
| *int2* | P0 | P1 | P2 | P4 | P7 | P12 |
| 2010 | NA | < LOD | < LOD | < LOD | 4.7 ± 4.6 | 5.2 ± 4.6 |
| 2012 | < LOD | 4.2 ± 3.9 | < LOQ | < LOD | 4.3 ± 3.9 | 7.1 ± 5.8 |
| Oct_13 | < LOD | < LOQ | < LOQ | 7.0 ± 5.9 | 5.6 ± 3.8 | 7.2 ± 6.1 |
| Feb_14 | < LOD | < LOQ | 6.0 ± 5.0 | 5.1 ± 3.3 | < LOQ | 5.8 ± 5.0 |
| Apr_14 | < LOD | < LOD | 5.1 ± 5.1 | 6.2 ± 6.0 | < LOQ | 6.7 ± 5.9 |
| Jul_14 | < LOD | < LOD | < LOQ | < LOQ | < LOQ | 5.5 ± 5.0 |
| *int3* | P0 | P1 | P2 | P4 | P7 | P12 |
| 2010 | NA | < LOQ | < LOQ | 5.3 ± 4.5 | < LOD | 8.0 ± 6.4 |
| 2012 | < LOD | 5.6 ± 4.4 | < LOQ | 5.9 ± 5.0 | 6.6 ± 5.5 | 8.4 ± 7.4 |
| Oct_13 | < LOD | 7.8 ± 6.9 | 7.2 ± 6.0 | 8.3 ± 7.3 | 8.0 ± 6.9 | 8.8 ± 7.6 |
| Feb_14 | < LOD | 7.0 ± 6.0 | 8.3 ± 6.8 | 8.3 ± 7.3 | 7.8 ± 6.2 | 7.9 ± 6.5 |
| Apr_14 | < LOD | 7.8 ± 7.4 | 7.3 ± 6.4 | 8.2 ± 7.3 | 6.8 ± 5.5 | 8.2 ± 7.2 |
| Jul_14 | < LOD | < LOQ | 6.4 ± 5.9 | 7.0 ± 6.8 | 5.7 ± 4.4 | 8.0 ± 7.5 |
| Total Bacteria (16S rDNA) | P0 | P1 | P2 | P4 | P7 | P12 |
| 2010 | NA | 10.8 ± 9.9 | 10.5 ± 9.2 | 11.1 ± 9.8 | 11.0 ± 10.0 | 12.3 ± 11.0 |
| 2012 | 10.3 ± 8.8 | 11.1 ± 9.8 | 11.6 ± 10.3 | 11.1 ± 9.8 | 11.1 ± 10.0 | 12.4 ± 10.9 |
| Oct_13 | 10.6 ± 9.3 | 10.7 ± 8.8 | 11.4 ± 10.4 | 11.6 ± 10.4 | 11.2 ± 10.0 | 11.7 ± 11.0 |
| Feb_14 | 10.5 ± 9.4 | 10.2 ± 9.2 | 11.6 ± 10.4 | 11.4 ± 10.3 | 11.4 ± 10.3 | 11.0 ± 10.0 |
| Apr_14 | 10.4 ± 9.2 | 11.5 ± 10.2 | 11.6 ± 10.1 | 11.9 ± 10.3 | 10.9 ± 9.6 | 11.8 ± 10.3 |
| Jul_14 | 10.3 ± 9.1 | 8.5 ± 8.1 | 11.3 ± 8.9 | 11.3 ± 10.0 | 11.2 ± 9.6 | 11.8 ± 10.7 |
| HF183 | P0 | P1 | P2 | P4 | P7 | P12 |
| 2010 | NA | < LOD | < LOD | < LOD | < LOD | 6.7 ± 5.6 |
| 2012 | < LOD | <LOQ | < LOD | < LOD | <LOQ | 6.4 ± 4.4 |
| Oct_13 | < LOD | <LOQ | <LOQ | 5.7 ± 4.8 | < LOD | 6.5 ± 5.1 |
| Feb_14 | < LOD | 5.0 ± 4.3 | 6.3 ± 5.4 | 5.8 ± 4.8 | <LOQ | 6.2 ± 4.4 |
| Apr_14 | < LOD | < LOD | < LOD | <LOQ | <LOQ | 5.8 ± 5.1 |
| Jul_14 | < LOD | < LOD | < LOD | < LOD | < LOD | 4.5 ± 4.4 |
| Total Bacteroidales | P0 | P1 | P2 | P4 | P7 | P12 |
| 2010 | NA | 5.4 ± 3.6 | 5.0 ± 4.1 | 5.6 ± 4.9 | 6.7 ± 4.9 | 9.1 ± 7.7 |
| 2012 | 4.7 ± 0.0 | 5.8 ± 4.3 | 6.4 ± 5.5 | 6.2 ± 5.3 | 6.2 ± 5.2 | 9.8 ± 9.0 |
| Oct_13 | 5.6 ± 4.9 | 8.3 ± 7.6 | 7.8 ± 6.4 | 9.2 ± 8.1 | 8.5 ± 7.2 | 9.9 ± 8.9 |
| Feb_14 | 5.4 ± 5.0 | 7.5 ± 6.2 | 9.5 ± 8.5 | 9.7 ± 7.9 | 8.5 ± 7.4 | 9.4 ± 7.8 |
| Apr_14 | 5.2 ± 5.0 | 8.0 ± 6.9 | 8.3 ± 7.3 | 8.7 ± 7.8 | 6.9 ± 5.6 | 10.4 ± 8.7 |
| Jul_14 | 4.1 ± 0.0 | < LOD | 6.7 ± 5.6 | 7.5 ± 6.2 | 6.4 ± 6.0 | 9.2 ± 8.3 |

NA: Not analyzed, <LOD: below limit of detection, <LOQ: below limit of quantification

Table S11. Assessment of partition changes over time or sampling site for the dominant genera recorded in Fig. 4.

| Genus | Year | | | | | | |  | Site | | | | | |
| --- | --- | --- | --- | --- | --- | --- | --- | --- | --- | --- | --- | --- | --- | --- |
|  | Kruskal  all data | 2010 | 2012 | Oct_2013 | Feb_2014 | Apr_2014 | Jul_2014 |  | Kruskal all data | P1 | P2 | P4 | P7 | P12 |
|  |  | Wilcoxon test | | | | | |  |  | Wilcoxon test | | | | |
| Actinobacteria/*Gaiella* | p<0.05 | a | a | ab | a | b | ab |  | p>0.05 | - | - | - | - | - |
| Actinobacteria/*Microbacterium* | p<0.05 | a | b | b | b | b | b |  | p>0.05 | - | - | - | - | - |
| Actinobacteria/*Mycobacterium* | p<0.05 | a | b | a | a | a | ab |  | p>0.05 | - | - | - | - | - |
| Actinobacteria/*Nocardioides* | p>0.05 | - | - | - | - | - | - |  | p<0.05 | a | a | a | a | a |
| Bacteroidetes/*Bacteroides* | p<0.05 | a | a | a | a | a | a |  | p>0.05 | - | - | - | - | - |
| Bacteroidetes/*Chryseobacterium* | p<0.01 | ac | a | b | b | bc | b |  | p>0.05 | - | - | - | - | - |
| Bacteroidetes/*Chryseolinea* | p<0.01 | ab | b | c | abc | ac | ab |  | p>0.05 | - | - | - | - | - |
| Bacteroidetes/*Cloacibacterium* | p<0.01 | ac | a | b | a | a | bc |  | p>0.05 | - | - | - | - | - |
| Bacteroidetes/*Macellibacteroides* | p<0.01 | a | ab | b | ab | a | ab |  | p>0.05 | - | - | - | - | - |
| Bacteroidetes/*Ohtaekwangia* | p<0.05 | ac | a | bc | ac | b | abc |  | p>0.05 | - | - | - | - | - |
| Bacteroidetes/*Paludibacter* | p<0.05 | a | a | b | a | b | a |  | p>0.05 | - | - | - | - | - |
| Bacteroidetes/*Prolixibacter* | p>0.05 | - | - | - | - | - | - |  | p<0.05 | a | a | ab | ab | b |
| Protebacteria/*Acidibacter* | p<0.01 | ab | a | b | a | b | a |  | p>0.05 | - | - | - | - | - |
| Protebacteria/*Anaeromyxobacter* | p>0.05 | - | - | - | - | - | - |  | p<0.01 | a | a | a | a | b |
| Protebacteria/*Cystobacter* | p<0.01 | a | a | b | b | b | b |  | p>0.05 | - | - | - | - | - |
| Protebacteria/*Ferribacterium* | p<0.05 | a | a | a | a | a | a |  | p>0.05 | - | - | - | - | - |
| Protebacteria/*Haliangium* | p<0.01 | ab | a | b | a | b | ab |  | p>0.05 | - | - | - | - | - |
| Protebacteria/*Massilia* | p<0.01 | a | b | bc | c | c | c |  | p>0.05 | - | - | - | - | - |
| Protebacteria/*Peredibacter* | p<0.05 | a | a | a | a | a | a |  | p>0.05 | - | - | - | - | - |
| Protebacteria/*Steroidobacter* | p<0.01 | a | a | ab | b | b | b |  | p>0.05 | - | - | - | - | - |

See Fig. 1 for position of the sampling sites and Table S1 for the main features of the sampling campaigns.

| Table S12. qPCR primers and probes used in this study | |  |  |  |  |
| --- | --- | --- | --- | --- | --- |
| Name | Sequence (5' - 3') | Tm(°C) | Amplicon size (bp) | Target | Reference |
| int1 |  |  |  |  |  |
| int1f2 | TCGTGCGTCGCCATACA | 56 | 67 | *int1* | (Ga*ze et a*l., 2011) |
| int1r2 | GCTTGTTCTACGGCCGTTTGA |  |  |  |
| int2 |  |  |  |  |  |
| int2qF | TTTACGCTGCTGTATGGTGC | 59 | 127 | *int2* | This study |
| int2qR | GGCTGTTTCTGCTTTTCCCA |  |  |  |  |
| int3 |  |  |  |  |  |
| int3qF | GCGGTCGAAATCCACATCC | 61 | 125 | *int3* | This study |
| int3qR | AGGTTCAGACGTTGCTTTCG |  |  |  |  |
| HF183 |  |  |  |  |  |
| HF183F | ATCATGAGTTCACATGTCCG | 60 | 81 | *rrnS* | (Seurin*ck et a*l., 2005) |
| HF183R | TACCCCGCCTACTATCTAATG |  |  |  |
| AllBac |  |  |  |  |  |
| AllBac296F | GAGAGGAAGGTCCCCCAC | 60 | 106 | *rrnS* | (Layt*on et a*l., 2006) |
| AllBac467R | CGCTACTTGGCTGGTTCAG |  |  |  |  |
| AllBac375Bhqr | FAM^a^-CCATTGACCAATATTCCTCACTGCTGCT-BHQ1 |  |  |  |  |
| ^a^FAM: 6-carboxyfluoresceine; BHQ1: Black Hole Quencher 1 | |  |  |  |  |

References: Gaze WH, Zhang L, Abdouslam *NA, et a*l. (2011) Impacts of anthropogenic activity on the ecology of class 1 integrons and integron-associated genes in the environment*. ISME* **J** 5: 1253-1261; Layton A, McKay L, Williams D, Garrett V, Gentry R & Sayler G (2006) Development of Bacteroides 16S rRNA gene TaqMan-based real-time PCR assays for estimation of total, human, and bovine fecal pollution in water*. Appl Environ Microbi*o**l** 72: 4214-4224; Seurinck S, Defoirdt T, Verstraete W & Siciliano SD (2005) Detection and quantification of the human-specific HF18*3 Bacteroid*es 16S rRNA genetic marker with real-time PCR for assessment of human faecal pollution in freshwater*. Environ Microbi*o**l** 7: 249-259.
